# Integrated strategies for mosquito-borne disease control: a comprehensive review with emphasis on repellents and China’s practices

**DOI:** 10.1186/s13071-026-07275-7

**Published:** 2026-03-10

**Authors:** Liping Shi, Sheng Huang, Yijing Shu, Xia Zhou, Xu Xu, Liping Liu, Qiang Fu

**Affiliations:** 1 Green Pharmaceutical Technology Key Laboratory of Luzhou City, Central Nervous System Product and Development Key Laboratory of Sichuan Province, School of Pharmacy, Southwest Medical University, Luzhou, 646000 China; 2 Ningbo Dayang Technology Co., Ltd, Ningbo, 315000 China; 3 Department of Biological Sciences, College of Biological and Environmental Sciences, Zhejiang Wanli University, Ningbo, 315100 China

**Keywords:** Mosquito-borne diseases, Mosquito control, Chemical repellents, Plant-based repellents, Formulation technology

## Abstract

Mosquito-borne diseases cause millions of deaths every year, imposing a heavy burden on global public health systems and socioeconomic development. In China, mosquito-borne diseases exhibit a pattern of imported cases leading to localized transmission, and in recent years, periodic outbreaks of related diseases have continued to erupt, posing a serious threat to public health security. Additionally, due to global warming, the suitable living range for mosquitoes is continuously expanding, increasing the frequency of outbreaks and the affected areas. Although substantial global resources have been invested in prevention and control research, a definitive solution to completely eradicate mosquito-borne transmission has not yet been established. This review systematically summarizes the three principal mosquito control strategies—mechanical, biological, and chemical—with a particular emphasis on chemical repellents. It delineates the structure-activity relationships, efficacy profiles, mechanisms of action, and toxicological evaluation results of major synthetic repellents. It also elaborates on the active constituents and action mechanisms of plant-based natural repellents, exploring how emerging formulation technologies can enhance the stability and duration of repellents. Moreover, together with China’s practical experience in mosquito-borne disease prevention and control, this review examines domestic research achievements and policy standards, ultimately constructing a theoretical framework to support the development of efficient, safe, and environmentally sustainable mosquito control solutions, thereby contributing to both national and global efforts in mosquito-borne disease prevention and control.

**Graphical Abstract:**

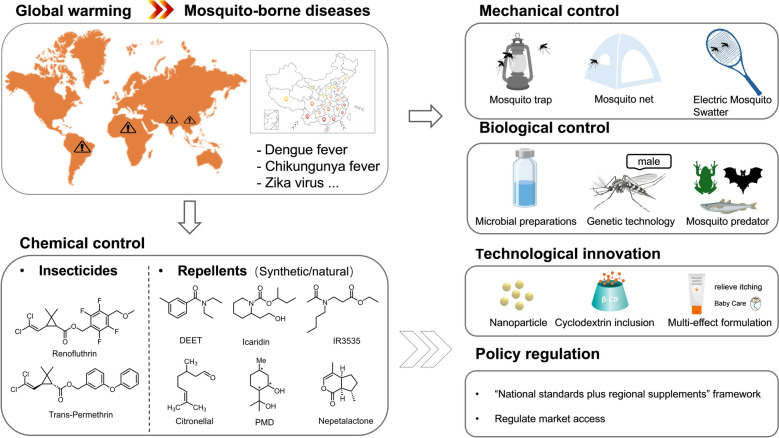

## Background

Vectors are living organisms capable of transmitting infectious diseases between humans or from animals to humans [1]. Worldwide, vector-borne infectious diseases account for 17% of all infectious diseases, and more than 700,000 people die annually from vector-borne disease [1]. With the impact of global climate change and other factors, a greater expansion is expected between 2050 and 2080, with about half of the global population (approximately 49%) at risk of virus transmission [2].

Among arthropods that transmit insect-borne diseases, mosquitoes are the most important vector of related diseases [3], and they are widely regarded as the deadliest animal on earth [4–6]. Beyond being a nuisance, mosquitoes act as blood-sucking insects that acquire pathogenic microorganisms from infected human or animal hosts through blood meals, subsequently transmitting the replicating pathogens to new hosts [1], causing serious public health threats worldwide. Currently, > 40 types of pathogens are transmitted by mosquitoes, including viruses such as dengue, yellow fever, chikungunya, Japanese encephalitis, and Zika virus disease, as well as protozoan parasites that cause malaria, which are the biggest contributors to the burden of vector disease transmission (Table 1) [6–10]. Each disease has distinct transmission vectors and endemic regions. Malaria is transmitted through the bites of *Anopheles* mosquito and is primarily endemic in sub-Saharan Africa and Southeast Asia [11]. Japanese encephalitis is transmitted by Tri-banded *Culex* mosquitoes and is primarily distributed across the Far East and Southeast Asia [12]. Zika virus disease is prevalent in tropical and subtropical regions worldwide, with widespread distribution in Africa, the Americas, Southeast Asia, and the Western Pacific. Both dengue fever and yellow fever are transmitted by *Aedes* mosquitoes, with dengue being especially prevalent in tropical and subtropical climates globally [12]. In recent years, global warming has significantly accelerated the spread of mosquito-borne diseases. Elevated temperatures facilitate faster mosquito reproduction in favorable environments, enabling vectors to invade previously unaffected areas [13]. Concurrently, the geographic range of mosquito natural hosts continues to expand, contributing to a sustained increase in disease incidence and triggering large-scale outbreaks that pose severe challenges to socioeconomic development [14, 15].

**Table 1:** Epidemiological trends of major mosquito-borne diseases

Vector-borne diseases	Main media	Global trends	Trends in China
Malarial	*Anopheles* genus	Primarily concentrated in South America and Southeast Asia[11]	Almost all new malaria cases are imported, concentrated in southeastern coastal and southwestern border areas[16]
Dengue fever	*Aedes aegypti*/*Aedes albopictus*	Found in tropical and subtropical climates worldwide[12]	Primarily distributed in southern regions such as Guangdong, Yunnan, and Zhejiang[17, 18]
Yellow fever	*Aedes aegypti*	It is primarily prevalent in tropical regions such as South America, Central America, and Africa and is also distributed across tropical countries in Asia[12]	China confirmed its first imported case of yellow fever in Beijing on 12 March 2016[17]
Japanese encephalitis	Tri-banded *Culex* mosquito	Primarily distributed in the Far East and Southeast Asia[12]	Except for Xinjiang, Tibet, and Qinghai, all other provinces (autonomous regions) have reported cases of Japanese encephalitis or localized outbreaks. Sichuan, Yunnan, Guizhou, Chongqing, and Guangxi are designated as high-risk areas for Japanese encephalitis[17]
Zika virus disease	*Aedes aegypti*	It is prevalent primarily in tropical and subtropical regions worldwide, with a widespread distribution across Africa, the Americas, Southeast Asia, and the Western Pacific region[12]	On 19 January 2016, Taiwan Province in China reported one imported case of Zika virus infection. Subsequently, on 6 February of the same year, the first imported case of Zika virus disease in mainland China was identified in Jiangxi Province[17, 19]
Chikungunya fever	*Aedes albopictus*/*Aedes aegypti*	The disease is primarily prevalent in Africa, Southeast Asia, and the Indian Ocean region, exhibiting a trend of sustained transmission or regional outbreaks in recent years[12]	China first reported imported cases in 2016. In 2024, imported cases and local transmission emerged in Guangdong, Guangxi, and Yunnan. In July 2025, an imported secondary cluster outbreak occurred in Foshan, Guangdong, with no neonatal-related cases identified[19]

**Fig. 1:**
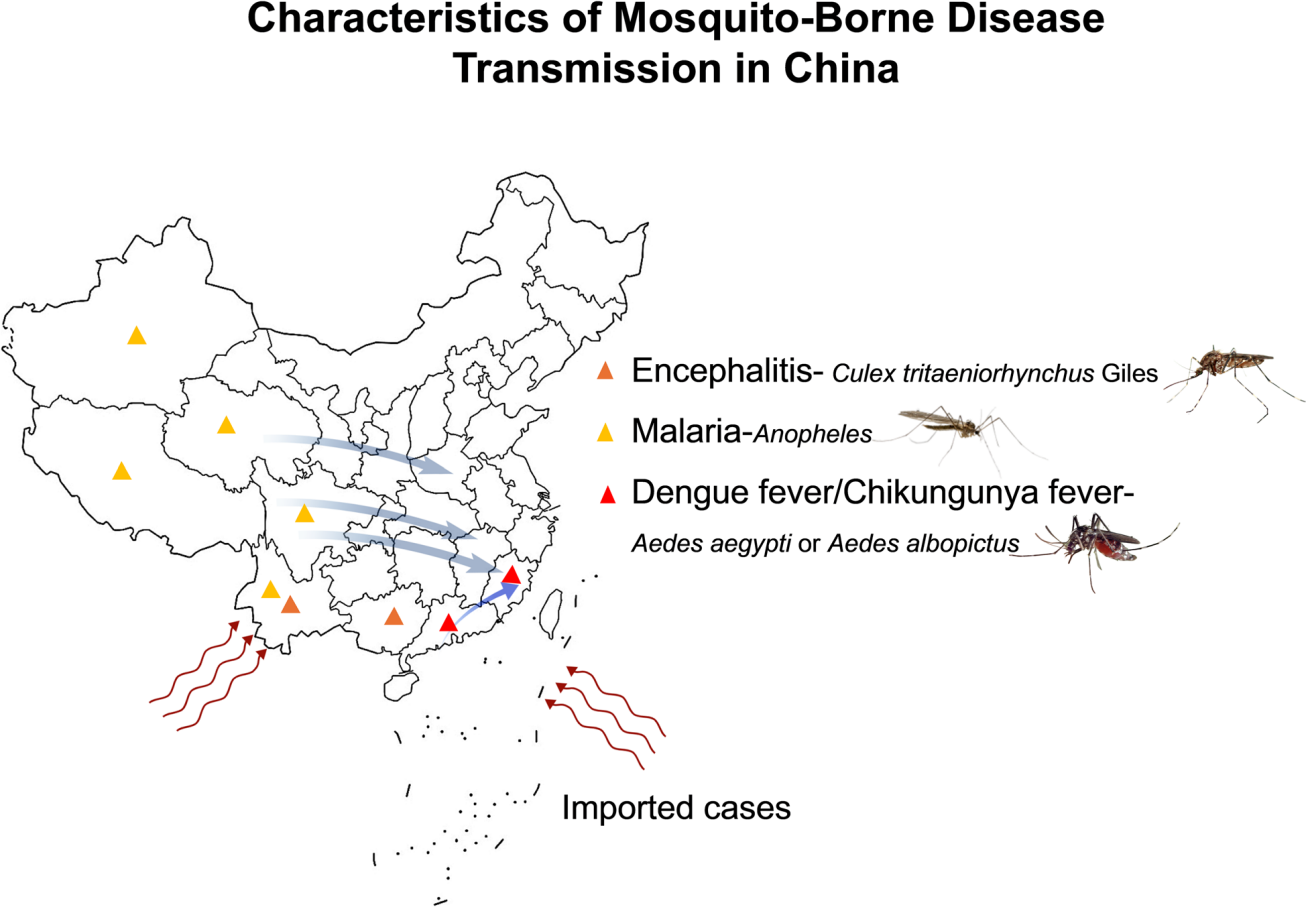
Characteristics of mosquito-borne disease transmission in China. Images were created with BioGDP.com[20]

In China, mosquito-borne diseases typically follow a pattern of imported cases leading to localized transmission, presenting a significant challenge to prevention and control efforts (Fig. 1). Malaria was once endemic in provinces such as Yunnan and Guangxi, with over 30 million annual cases. Currently, there are no indigenous cases, and all newly reported infections are imported [16]. Japanese encephalitis is widely distributed across the country, except for Xinjiang, Tibet, and Qinghai. High-incidence areas include Sichuan and Yunnan provinces, although the overall prevalence has stabilized in recent years. Regarding imported diseases, the primary vectors are *Aedes* mosquitoes, which pose elevated higher risks in southern provinces such as Yunnan and Guangdong [17]. Diseases such as dengue fever and chikungunya have shown trends of transmission or outbreaks in recent years [17]. In 2024, Guangdong and other provinces experienced imported dengue cases and local transmission, with Guangdong alone registering > 10,000 cumulative cases during the period [18]. Climate warming has extended mosquito-suitable habitats northward, leading to dengue's expansion to provinces such as Zhejiang [17]. The first imported cases of yellow fever, Zika virus disease, and chikungunya in China were all reported in 2016. In 2025, Foshan (Guangdong) also experienced a cluster outbreak of chikungunya virus [19].

Driven by climate warming and trade globalization, mosquito-borne diseases are expanding globally, with novel pathogens continually emerging. In this context, China, characterized by a unique “imported-to-domestic transmission” pattern, has become a critical region in the landscape of mosquito-borne disease control. Regional variation in geographical environments and climatic conditions, mosquito species compositions, and ecological patterns, as well as population lifestyles and behavioral patterns, presents substantial challenges for disease surveillance and intervention. This diversity underscores the need for region-specific mosquito control strategies and locally adapted solutions. By systematic review of major mosquito vector control approaches, the properties of synthetic and plant-based repellents, strategies for the development of new repellent formulations, and China’s policies, this review aims to provide robust theoretical and technical support for targeted mosquito-borne disease control. This, in turn, will promote upgrading of the domestic mosquito repellent industry and strengthen public health security. Furthermore, it offers valuable insights from China’s experience to inform global mosquito control efforts, having profound socioeconomic significance (Table 2).

**Table 2:** Classification and comparison of core characteristics of synthetic mosquito repellents

Structural classification	Core representative compounds	Structural formula	Advantages and disadvantages
Amides	N,N-diethyl-m-toluamide (DEET)		Broad-spectrum protection, with concentrations of 10%–30% offering potent and long-lasting efficacy [21]; presents safety risks (including dermatitis and neurological damage) [22–24]; possesses a pungent odor and greasy texture, diminishing sun protection effectiveness[25, 26]; exhibits corrosive properties and contributes to environmental pollution[27, 28]
	N, N-diethylphenylacetamide (DEPA)		Broad-spectrum mosquito repellent with good protective effects[29], low toxicity, non-irritating, and low cost[20, 30]; Its repellent mechanism is unclear[31]
Piperidine class	sec-Butyl 2-(2-hydroxyethyl)piperidine-1-carboxylate(icaridin)		15%-20% concentration protection for 8–10 h[21, 32]; Colorless, odorless, non-greasy, low toxicity, biodegradable, does not damage materials[32, 33]; expensive and can irritate sensitive individuals[34, 35]
	(1S,2S)-2-methylpiperidine-3-cyclohexene-1-carboxamide(SS220)		Effective against *Aedes* and *Anopheles* mosquitoes in Egypt, with effects comparable to 33% DEET[36]; It has low toxicity and irritation, with low volatility [119–122]; the separation of active isomers is costly, and it is not registered [70]
Esters	Ethyl butylacetylaminopropionate (IR3535)		IR3535 (20%) provides 7–10 h of protection against *Culex* and *Aedes* mosquitoes, and 3–4 h against *Anopheles* mosquitoes, which is shorter than for DEET [37]. It is low in toxicity, mild in nature, and moisturising, making it suitable for children and pregnant women [23, 38]; The pure substance is viscous, prone to irritate skin, and may damage fabrics[21]
	Dimethyl 1,2-phthalate(DMP)		It possesses repellent properties against *Aedes* mosquitoes, although its efficacy is inferior to that of DEET and requires higher application concentrations[39]; characterized by low toxicity and absence of skin irritation; it may serve as a solvent[31, 40]; The product has since been withdrawn from the market[41]

## Mosquito control measures

Despite extensive research efforts, most major mosquito-borne viruses have no effective vaccines and targeted therapies, with the exception for vaccines against Japanese encephalitis and yellow fever [42]. Given the global and regional threats posed by mosquito-borne diseases, current prevention and control strategies can be categorized into mechanical, biological, and chemical approaches. Mechanical control emphasizes physical barriers; biological control prioritizes eco-friendly interventions; biological control focuses on environmentally friendly interventions; chemical control (particularly repellents) remains the core strategy due to its high efficacy. Collectively, these three approaches constitute an integrated mosquito-borne disease control system [43].

### Mechanical control

**Fig. 2:**
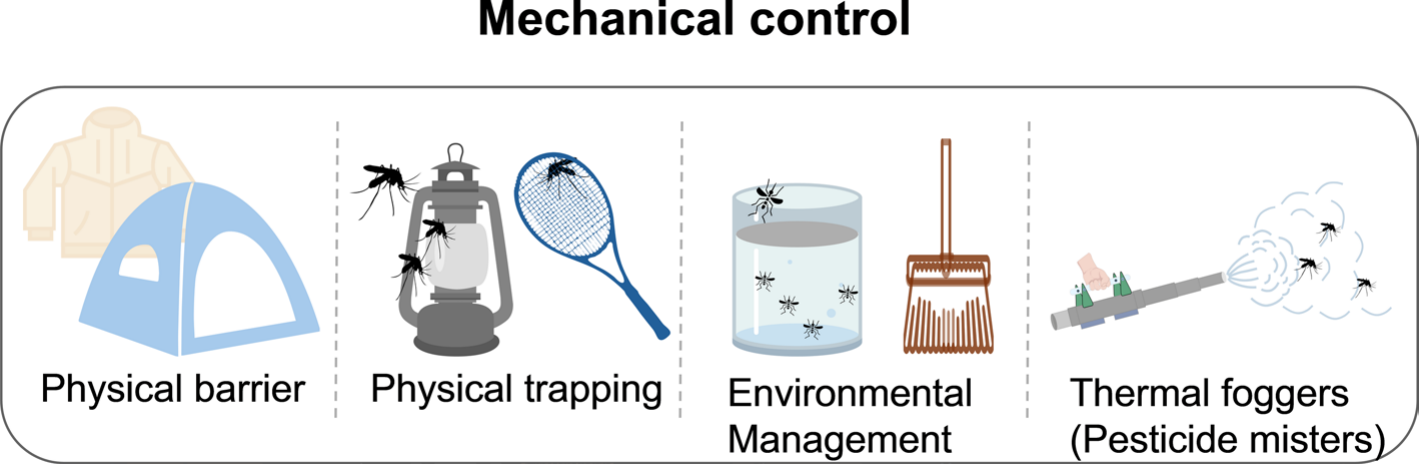
Mechanical control. Mosquito control is achieved via measures such as physical barriers, physical trapping, environmental management, and the combined use of chemical and physical methods. Images were created with BioGDP.com[20]

Mechanical mosquito control methods emphasize physical intervention and are generally categorized into three main types (Fig. 2): (i) the use of physical barriers, such as screens, mosquito nets, and protective clothing (e.g. light-colored, long-sleeved garments) to prevent mosquito bites; (ii) physical trapping and killing techniques, including mosquito traps and electric swatters, which aim to eliminate adult mosquitoes directly; (iii) environmental management approaches that disrupt mosquito breeding habitats, thereby reducing population density [44–48]. However, with the advancement of ecological research, concerns regarding the indiscriminate application of mechanical mosquito control methods have emerged. For instance, thermal fogging in Sri Lanka [49] and pyrethroid fogging in Malaysia [50], although chemically driven, are often implemented via mechanical means, and they have been shown to cause extensive non-target insect mortality. Affected groups include Diptera, Hymenoptera, particularly pollinating insects such as stingless bees, and Lepidoptera. Such interventions may also disrupt species composition and interfere with local food web dynamics.

**Fig. 3:**
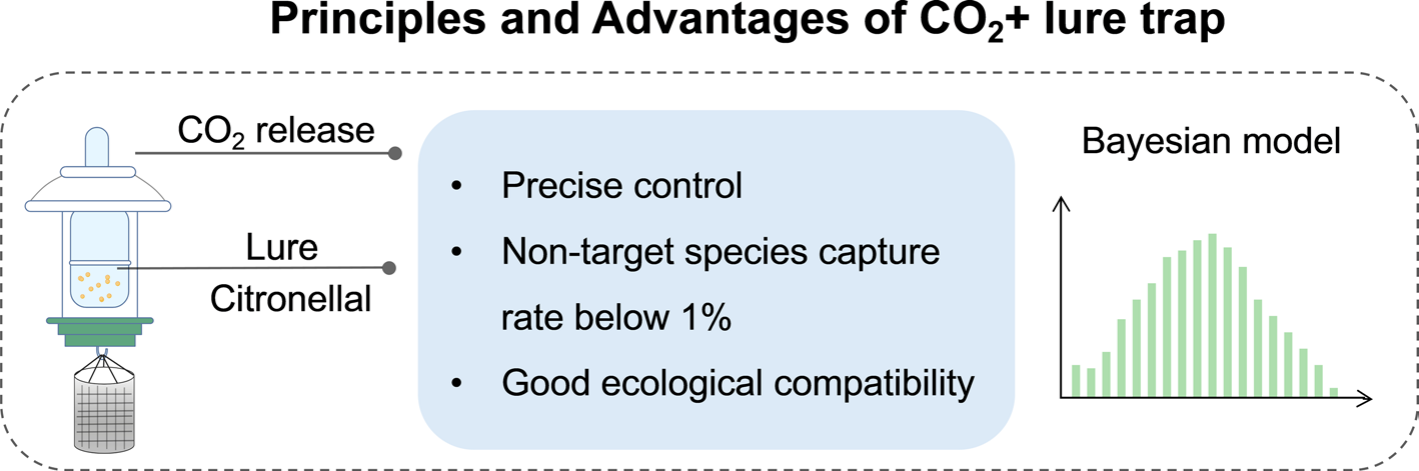
Principles and advantages of the CO^2^ + lure trap. Images were created with BioGDP.com[20]

Therefore, mechanical mosquito control systems must prioritize precision targeting while minimizing impacts on non-target species. In China, pilot programs in Guangdong and Yunnan provinces targeting the *Aedes albopictus* mosquito—the primary vector of dengue fever—have implemented locally developed traps utilizing carbon dioxide and citronella traps (Fig. 3). These traps have demonstrated 30% higher capture efficiency than imported equipment. Owing to their more targeted attractants, these devices result in < 1% incidental capture of local pollinators like bees and butterflies, demonstrating markedly improved ecological compatibility compared with traditional devices [51]. Operations avoid daytime periods when non-target species are active, focusing on localized areas to minimize large-scale mechanical spraying. Concurrently, Bayesian models and other methods are employed to routinely monitor non-target species [52, 53], facilitating the integration of environmental management into a multi-tiered control system that enables dynamic adjustments aimed at minimizing ecological impacts.

### Biological control

In recent years, the application of biological control strategies has gradually increased. These include the use of *Bacillus*-based microbial formulations, genetically modified entomopathogenic fungi, genetically modified mosquito (GMM) technologies, the sterile insect technique (SIT), and biological control using natural predators [43].

**Fig. 4:**
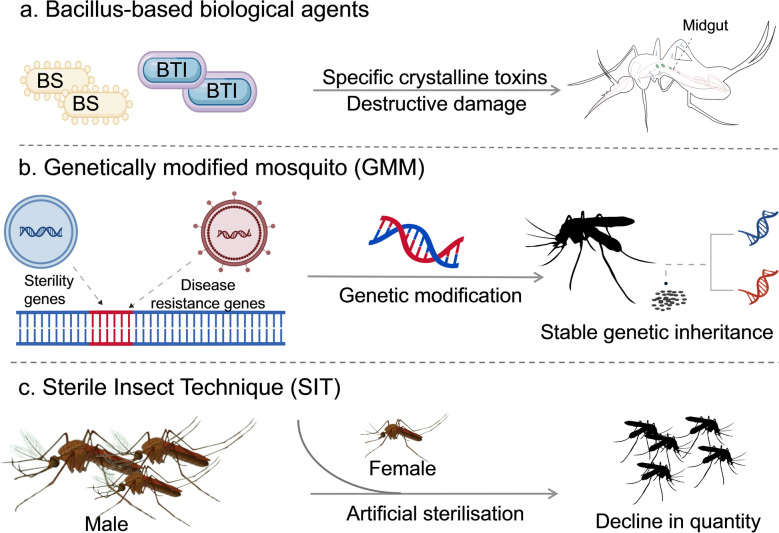
Biological control measures and principles. **a** *Bacillus*-based biological agents—typified by *Bacillus thuringiensis* subsp. * israelensis* (Bti) and *Bacillus sphaericus* (Bs)—synthesize species-specific crystalline toxins. These toxins disrupt the structural and physiological integrity of midgut epithelial cells in mosquito larvae, inhibiting nutrient absorption and metabolism and ultimately causing larval death. **b** Gene editing technologies and genetically modified mosquito (GMM) systems utilize gene drive mechanisms to rapidly propagate target modified genes (e.g. pathogen-resistance or sterility genes) within mosquito populations. By reducing the effective population size or impairing disease transmission capacity, these strategies substantially curb the spread of mosquito-borne diseases such as malaria and dengue fever. **c** The Sterile Insect Technique (SIT) operates through three core steps: large-scale artificial rearing of male mosquitoes, inactivation of their germ cells via gamma irradiation or chemical induction, and targeted release into natural environments. These sterile males compete with wild males for female mating opportunities, lowering the effective mating success of wild females and thereby enabling long-term regulation of mosquito population density. Images were created with BioGDP.com[20]

*Bacillus*-based biological agents represent (Fig. 4a) core biological pesticides for the control of mosquito larvae, with *Bacillus thuringiensis* subsp. *israelensis* (Bti) and *Bacillus sphaericus* (BS) being the most widely used. Bti produces a specific crystalline toxin that disrupts the midgut of mosquito larvae, ultimately leading to their death. It is effective against larvae of multiple mosquito genera, including *Culex*, *Aedes*, and *Anopheles*, but is non-toxic to mammals and aquatic organisms. In Burkina Faso, the widespread application of the *Bacillus thuringiensis* larvicide led to a marked reduction in mosquito bites over a 3-year period [54]. In parallel, Weiguo Fang et al. [55] discovered an ecological strategy in which *Beauveria bassiana* attracts insect hosts by releasing longifolene, thereby promoting spore dispersal. Ramirez et al. [56] evaluated the larvicidal efficacy of two *Beauveria bassiana* spores against *Aedes aegypti* larvae. Their findings indicated that conidia exerted more rapid insecticidal activity, whereas chlamydospores demonstrated a delayed effect. The combined application of both propagules has been shown to enhance overall mosquito control efficacy. Regarding the emergence of drug-resistant *Aedes albopictus* populations in multiple regions in China, the M2 strain of *Beauveria bassiana* achieved a lethality rate of 88% against resistant mosquito populations. Notably, no resistance was observed after three consecutive generations of continuous application [57]. The Institute of Zoology, Chinese Academy of Sciences, has developed a phage-based mosquito control strategy, employing bacteriophages as the core agent to reduce population density by inhibiting larval development [58].

Advances in genetic manipulation technology and the deployment of genetically modified mosquitoes (GMMs) (Fig. 4b) have become prominent in the field of mosquito vector control [59]. It achieves mosquito vector control through gene drive technology, rapidly spreading modified genes—such as disease resistance or sterility genes—within mosquito populations. This strategy effectively suppresses mosquito populations and reduces the transmission of mosquito-borne diseases such as malaria and dengue fever [60, 61]. Xuejiao Xu et al. [62] have successfully engineered a “homing suppression-driven system.” The modified mosquitoes exhibit recessive sterility, with homozygotes displaying hermaphroditic traits and virtually no detectable resistance genes. This breakthrough offers significant insights into developing highly effective and environmentally sustainable tools for mosquito vector control, thereby breaking the malaria transmission chain. However, GMMs involve potential risks to non-target species, ethical concerns, and regulatory challenges, including unintended ecological impacts, potential misuse of the gene drive technology, and issues related to animal welfare. Therefore, further research is required to assess the safety and efficacy of GMM, and robust regulatory frameworks must be established to ensure their safe deployment [63]. To date, China has carried out limited gene editing research on *Aedes albopictus* mosquitoes in laboratory settings, with no field releases having been conducted. Additionally, the enactment of the Administrative Measures for the Safety of Biotechnology Research and Development provides a legal foundation for regulating the use of genetically modified organisms in scientific and public health contexts [64].

The Sterile Insect Technique (SIT) (Fig. 4c) is a biological vector control method involving the mass rearing, sterilization, and release of male insects into the wild to compete with wild males for mating opportunities [65]. This reduces opportunities for wild females to mate with fertile males, leading to a gradual decline in the target insect population [66]. Although the introduction of mosquito predators does not fall within the conventional definition of biological control, it often serves as a vital supplementary measure within biological control systems. When used in conjunction with microbial agents, such ecological interventions can produce synergistic effects that enhance overall control efficacy. For instance, the introduction of mosquito-eating fish (such as *Poecilia reticulata*) into water bodies like courtyards and ponds has been employed for the biological control of mosquito larvae. At ambient temperatures, mosquito-eating fish such as *Betta splendens* and *Aplocheilus panchax* can achieve predation efficiencies of 75% and 72.3%, respectively, against *Aedes albopictus*. In practice, suitable fish species should be selected based on local water temperatures to effectively control mosquito populations [67]. In southern China, introducing mosquito-eating fish into ponds and rice paddies has increased larval mortality rates of *Anopheles sinensis*, without significantly affecting native fish populations. This practice has become a complementary measure in mosquito vector control in rural regions of southern China [68].

Current biological control methods can effectively suppress mosquito populations and reduce disease transmission while ensuring environmental safety and minimizing harm to non-target organisms. However, several challenges persist, including limitations in storage, transportation, and field stability and potential ecological risks. Moreover, some emerging technologies still require further regulatory clarification. Through continued technological optimization and the implementation of standardized management practices, biological control approaches are expected to become a central component of integrated mosquito vector control systems.

### Chemical control

**Fig. 5:**
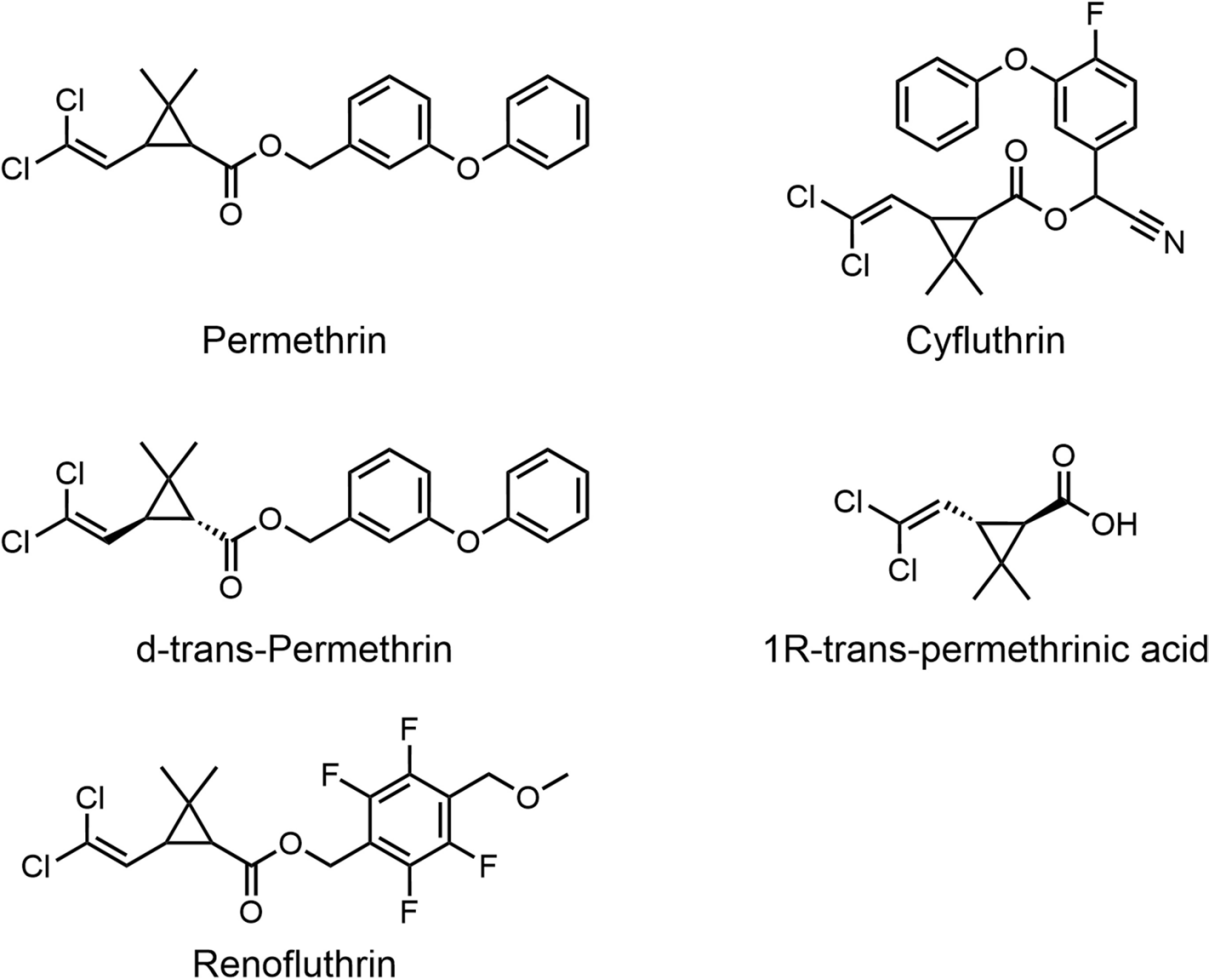
The chemical structures of permethrin, d-trans-permethrin, cyfluthrin, renofluthrin, and 1R-trans-permethrinic acid

**Fig. 6:**
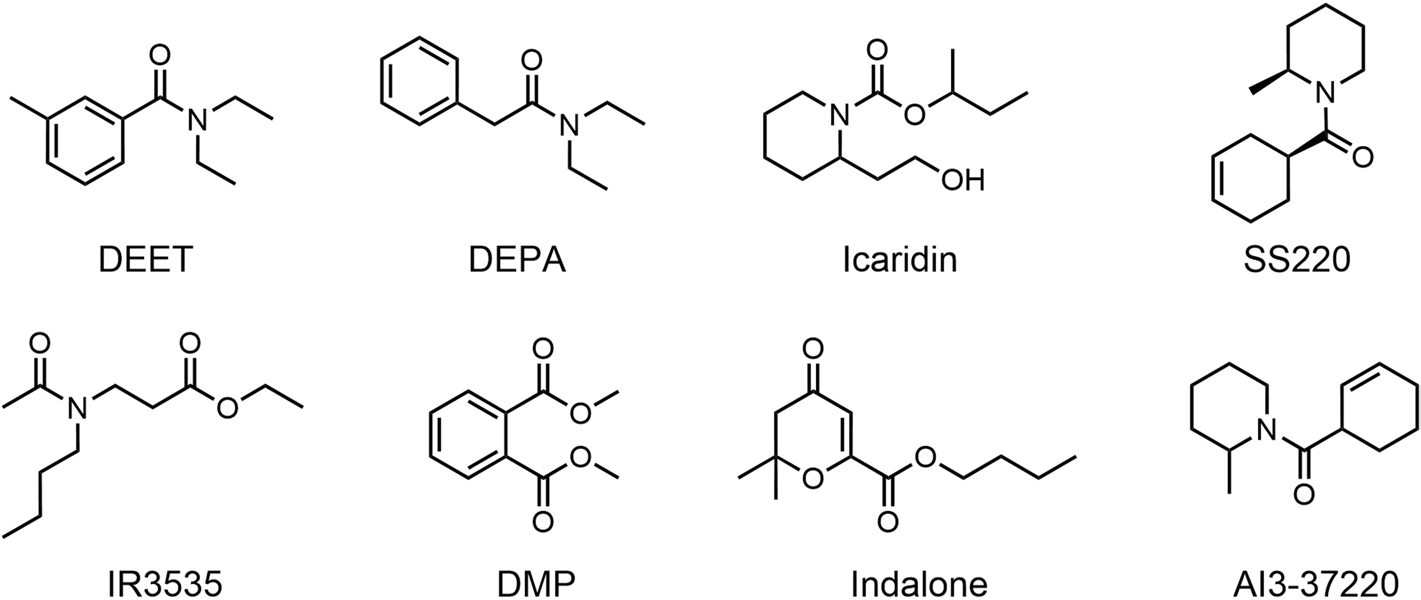
Chemical structure of synthetic mosquito-repellent compounds, including DEET, DEPA, icaridin, SS220, IR3535, DMP, indalone, and AI3-37,220

Chemical control remains one of the oldest and most effective control measures, continuing to play a critical role in contemporary vector control programs [69]. Traditional mosquito repellents often involve the application or combustion of plant materials, such as mugwort and hemp leaves, to release plant oils, smoke, or tar with repellent properties [70]. For example, burning wood and leaves (such as mango, coconut, ginger, and betel leaves) outdoors can eliminate 57% to 75% of mosquitoes [71]. However, smoke-based mosquito repellents require prolonged combustion and are generally limited to outdoor use. Their application in indoor environments poses health risks and fire safety concerns. Consequently, people have begun exploring alternative safe and effective mosquito repellent methods, with insecticides and repellents emerging as the primary focus. Among commonly used insecticides, permethrin (Fig. 5) is one of the most representative compounds [31]. However, with the overuse of insecticides, mosquitoes have become resistant to them, and their extensive use also has adverse effects on non-target organisms and the environment, limiting their application [34, 72]. The repellents are divided into natural plant and chemical synthetic repellents; both are highly efficient, fast, and convenient, so they have been widely used [69]. Common chemical repellents such as DEET, icaridin, and IR3535 (Fig. 6) are widely formulated into various commercial repellent products, offering reliable and effective protection. In contrast, plant-based repellents—primarily composed of essential oils such as citronella (from *Cymbopogon* spp.) and lemon eucalyptus (from *Corymbia citriodora*)—have gained increasing popularity in recent years due to their environmentally friendly properties and pleasant fragrance, making them attractive to consumers seeking natural alternatives [3, 31].

An ideal mosquito repellent must achieve a balance among broad-spectrum efficiency, prolonged protection, human safety, and environmental compatibility: it should be effective against multiple mosquito species, provide long-lasting repellency, cause no irritation to human skin, and pose no harm to the ecological environment [69]. Since the introduction of DEET in the 1950s, chemical repellents have long dominated the market for tropical mosquito protection [73]. However, current chemical and natural repellent formulations remain insufficient in fully meeting the criteria for an ideal repellent. Addressing this limitation requires a more comprehensive understanding of repellent categories and their underlying mechanisms of action. The following section provides a systematic analysis of insecticide and representative chemical repellents, examining their molecular structure, mechanisms of mosquito-repellent action, and associated safety concerns. It will also focus on elucidating the active ingredient compositions of plant-based essential oils, including citronella, lemon eucalyptus, and catnip, and their repellent mechanisms through olfactory receptor interactions and visual interference effects. Furthermore, it will review recent advancements in formulation technologies across both chemical and botanical repellent categories, offering guidance for the development of mosquito-repellent products adapted to China’s regional ecological and epidemiological conditions.

### Insecticide

**Fig. 7:**
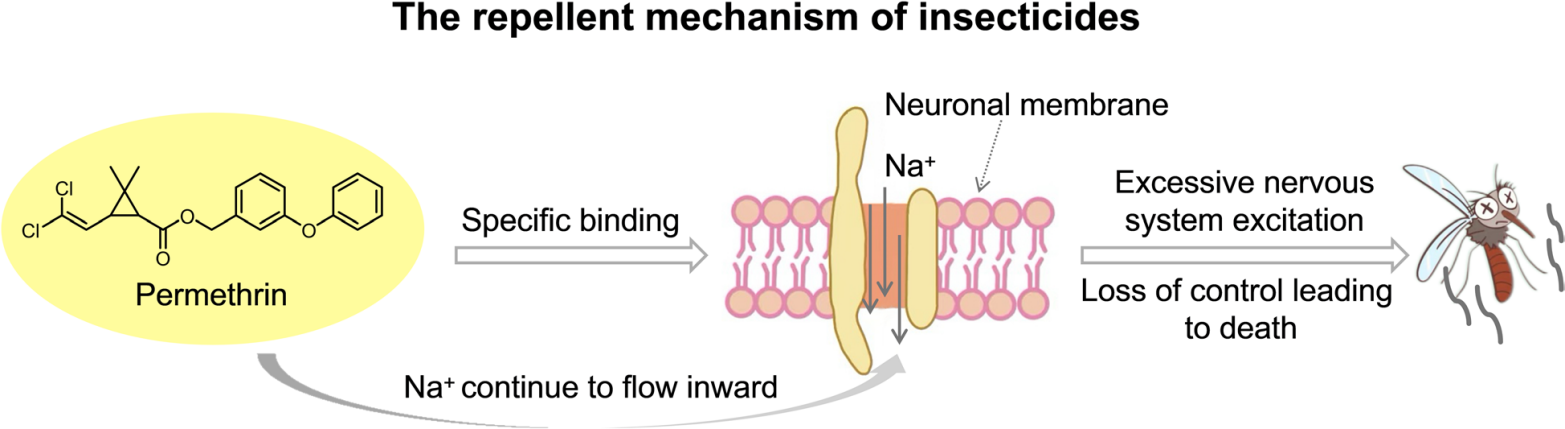
The repellent mechanism of insecticides. The core mechanism of this class of insecticide involves targeting voltage-gated sodium channels in mosquito neurons. By disrupting neural signal transmission, they induce sustained inward sodium influx, ultimately causing excessive excitation and death in the mosquito. Images were created with BioGDP.com[20]

Pyrethroid insecticides are synthetic analogs of natural pyrethrin, which is extracted from the silver-leaved chrysanthemum (*Tanacetum cinerariifolium*). They serve as essential tools in mosquito-borne disease control by disrupting host-seeking behavior in mosquitoes (Fig. 7) [74–76]. Among these compounds, volatile pyrethroid insecticides demonstrate strong potent insecticidal activity even at low concentrations. However, due to prolonged and widespread use, mosquito resistance has significantly increased worldwide [77]. Concurrently, increasing concerns have been raised regarding the potential human health risks associated with pyrethroid insecticides, including neurotoxicity, genotoxicity, and the induction of asthma—particularly among vulnerable populations such as infants, young children, and pregnant women [78–80]. Recent studies confirm that prenatal exposure to cyfluthrin (Fig. 5) causes neurodevelopmental toxicity in offspring, leading to cognitive impairments during both early childhood and adulthood [81]. Notably, these adverse effects exhibit long-term persistence. Moreover, trace residues of pyrethroid insecticides cause persistent ecological and societal harm through environmental migration and biological enrichment. In response to these concerns, several countries have begun to gradually phase out the use of cyfluthrin and related compounds on an annual basis [82, 83].

To address limitations associated with pyrethroid insecticides, significant progress has been made in the development of novel mosquito insecticidal active ingredients. Jeffrey et al. [84] focused on 1R-trans-permethrinic acid (Fig. 5) and a series of ester compounds containing small alkyl substituents, including n-propyl, n-butyl, n-pentyl, cyclobutyl, and cyclopentyl, which have demonstrated optimal performance. These compounds exhibited excellent insecticidal efficacy against both insecticide-susceptible Orlando (OR) and pyrethroid-resistant Puerto Rico (PR) strains of *Aedes aegypti*. Preliminary trials further indicate that these small-molecule alcohol esters exhibit synergistic effects when combined with aniline compounds and pyrethroid acid derivatives, thereby offering a promising strategy for controlling insecticide-resistant mosquitoes.

India’s domestically developed and patented novel pyrethroid compound, renofluthrin (Fig. 5) (active ingredient “renofluthrin”) is a specific isomer mixture containing 90%–99.90% (1R,3S) and (1S,3R) isomers); it exhibits dual insecticidal and repellent activity [85]. Even at one-quarter the concentration of *d*-*trans*-permethrin (Fig. 5) commonly used in mosquito coils in India, renofluthrin demonstrates superior and rapid insecticidal activity owing to its high vapor pressure and strong volatility. Moreover, it exhibits good compatibility with existing mosquito coil and household insecticide formulations and has potential for use in the development of novel delivery systems aimed at enhancing stability and controlled release. However, data concerning its environmental degradation, ecotoxicological characteristics, and aquatic toxicity remain unavailable. Regarding human health risk classification, cyfluthrinis is designated as Category III (high risk), indicating that strict safety precautions and risk mitigation strategies are necessary in practical applications [86].

However, these novel ingredients still present notable limitations. The small alcohol ester compounds developed by Bloomquist’s team have yet to demonstrate sufficient technical maturity for large-scale applications or long-term safety assurance. The manufacturing use product (MUP) of permethrin is only available in emulsifiable concentrate (EC) formulation. Given its narrow solubility range, this significantly limits its application in water-based formulations such as sprays. Overall, while novel mosquito insecticidal active ingredients demonstrate core advantages in efficacy enhancement and resistance management, they require further validation regarding formulation compatibility, industrial scalability, and long-term safety. Future research must adopt a more systematic and integrative approach to fully realize their potential while ensuring manageable risk profiles.

## Chemically synthesized mosquito repellent

**Fig. 8:**
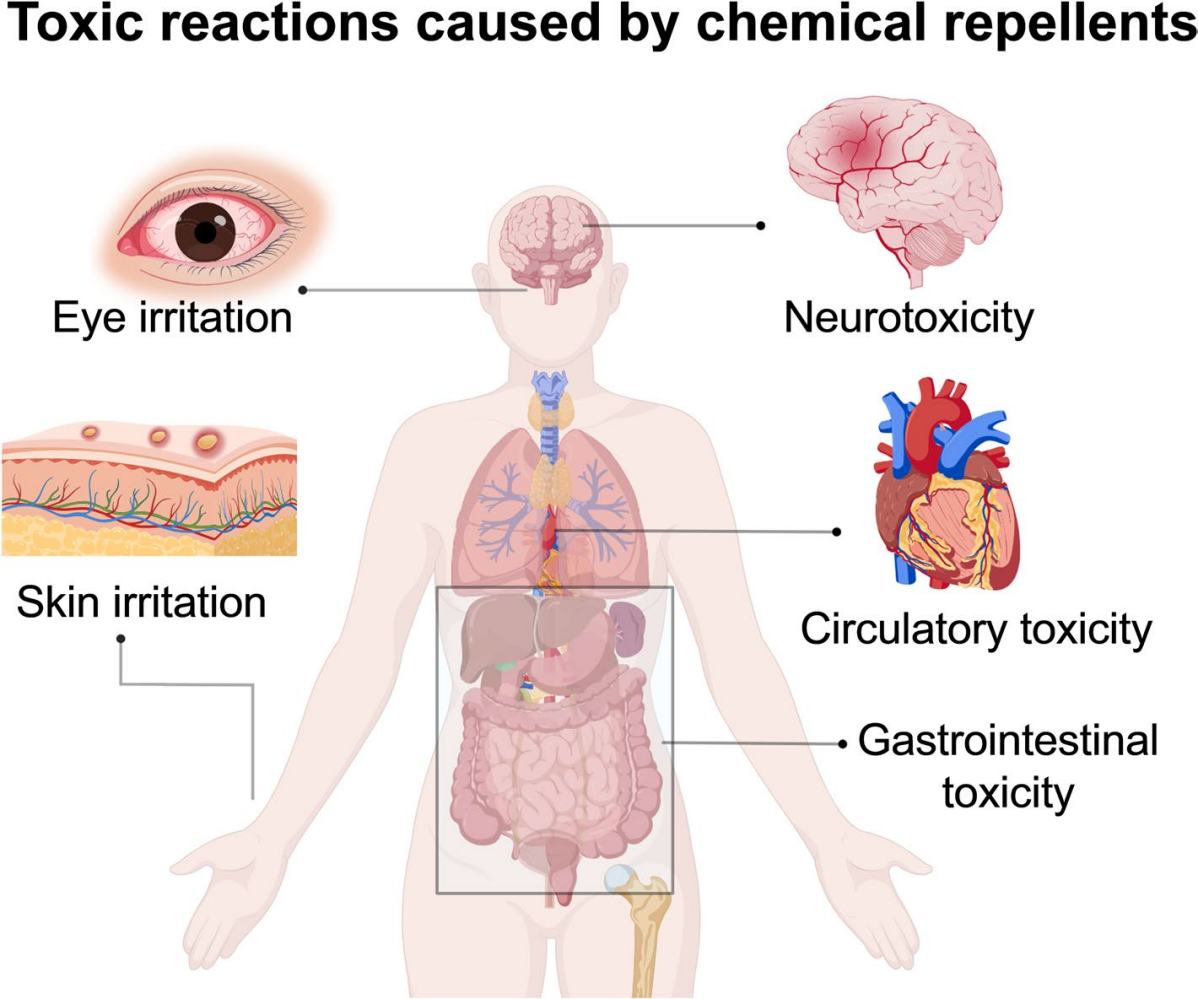
Toxic reactions caused by chemical repellents. Images were created with BioGDP.com[20]

Chemically synthesized mosquito repellents comprise synthetic chemicals that repel mosquitoes. The most widely used synthetic repellents are DEET and novel repellents developed from chemical structures such as amides, piperidine, diols, and phthalates, including hydroxypiperidines and mosquito repellents[73]. Consumers like chemical mosquito repellents because they are long- lasting, have a strong repellent effect, and are easy to carry. However, chemical mosquito repellents have some safety problems (Fig. 8) and are not environmently friendly, leading to some application restrictions.

### Amide-based repellents

#### N,N-diethyl-m-toluamide (DEET)

N,N-diethyl-m-toluamide (DEET, as shown in Fig. 6) is a broad-spectrum chemical synthetic mosquito repellent discovered by the US Department of Agriculture in 1946; it was then developed by the US Army and introduced to public use in 1957. It is by far the most widely used and effective “universal” mosquito repellent worldwide, with a strong and lasting repellent effect. It is the “gold standard” mosquito repellent [27, 87].

**Fig. 9:**
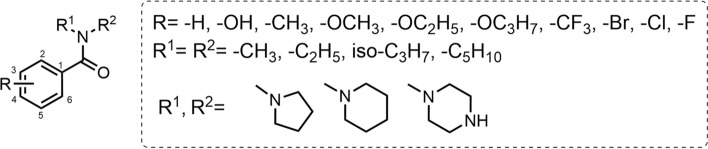
The structure-activity relationship of DEET

DEET's structure is derived from the structural frameworks of diethylamine and aromatic acids (Fig. 9). When substituent R^1^ and R^2^ are methyl, ethyl, or isopropyl, ethyl-substituted compounds exhibit greater mosquito-repellent activity. However, when the 3-position of the benzene ring is substituted with chlorine, methyl-substituted R^1^ and R^2^ show better activity. R^1^ and R^2^ can also form pyrrole, piperidine, or piperazine rings with the nitrogen atom. Piperazine ring formation leads to a complete loss of activity, while pyrrole or piperidine ring formation yields mosquito-repellent activity comparable to that of DEET. A substituent on the benzene ring significantly affects mosquito-repellent activity. These ring substituents may include hydrogen, hydroxyl, methyl, alkoxy, trifluoromethyl, and halogens (fluorine, chlorine, bromine), among other groups. Compounds with a methyl or hydrogen substituent on the benzene ring show higher mosquito-repellent activity; methyl substitution exhibits the most pronounced activity, with no decisive impact of substituent position on activity. For a methoxy substituent on the benzene ring, ortho-substitution enhances mosquito-repellent activity, whereas para-substitution is ineffective. If the benzene ring bears at least one fluorine-containing substituent, the compound consistently demonstrates some degree of mosquito-repellent activity. When the benzene ring is substituted with methyl at the 3-position and fluorine at the 5-position, the complete effective protection time at a 1% concentration exceeds that of DEET by 1 h [88–92].

**Fig. 10:**
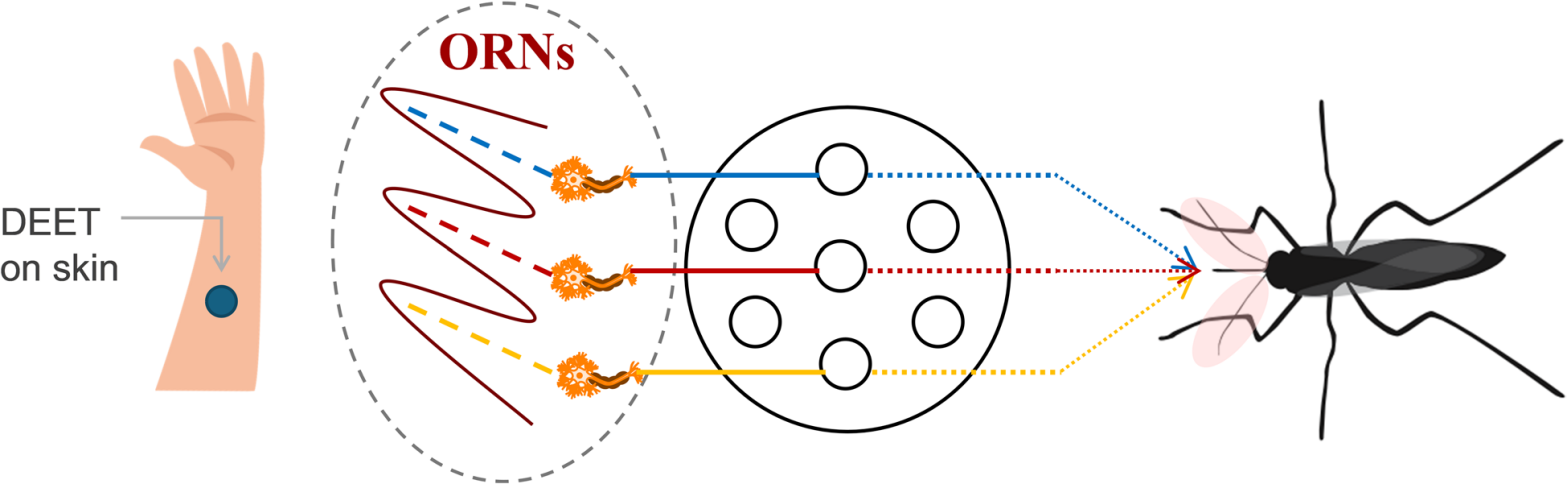
DEET's exact mechanism of action. ORNs (olfactory receptor neurons) are key components of the mosquito olfactory system. They can detect odor molecules in the air, convert them into electrical signals, and then transmit these signals to the mosquito’s central nervous system, assisting mosquitoes in behaviors such as host location, nectar seeking, and spawning site identification

Although DEET's exact mechanism of action (Fig. 10) remains incompletely understood, its elucidation is critical for the development of more effective mosquito repellents [93]. Current hypotheses suggest that DEET exerts its effects through molecular targets such as odor receptors (ORs) and ion receptors (e.g. Ir40a), functioning by either masking host odors or forming a vapor-phase barrier that prevents mosquitoes from detecting human skin [93–96]. DEET may interfere with the detection of host-derived volatiles, thereby impairing the mosquito’s ability to locate its prey [93]. Ali et al. [97] demonstrated that synthetic repellents do not directly activate Orco/OR by calcium imaging and behavioral experiments but chemically interact with OR ligands to prevent them from reaching the mosquito antennae.

As the gold standard mosquito repellent, DEET has long-term and lasting mosquito-repellent ability, and a formula containing 4%-100% DEET can be directly used on the skin to eliminate mosquitoes [70]. In most practical scenarios, products containing 10%–30% DEET should provide adequate protection [21]. The repellent efficacy and duration generally increase with concentration, although this effect plateaus at approximately 50% DEET [25]. Although many other repellents have been developed, such as IR3535, icaridin, and PMD (*para*-menthane-3,8-diol), they are not as effective as DEET. For example, Mark et al. [98] conducted arm-in-cage experiments to evaluate mosquito-repellent formulations containing various DEET concentrations. The results show that when the DEET concentration is 23.8%, the protection against mosquito bites lasts the longest, and the average complete protection time is 301.5 min. In comparison, formulations containing IR3535 outperformed those with plant-based oils such as citronella and cedar [98]. Due to the powerful repellent effect of DEET, it is listed as the preferred mosquito repellent in the Zika virus prevention and control guidelines [87].

Despite its favorable safety profile and strong protective efficacy against mosquitoes, DEET poses potential risks of toxicity, adverse reactions, and ecological harm, particularly when misused or over-applied [27]. Although the US Environmental Protection Agency (EPA) conducted comprehensive reviews of DEET’s safety and toxicity in 1998 and 2014 [99] and concluded that there is no significant risk when it is used at recommended doses, concerns remain regarding its potential adverse effects [22]. However, prolonged or excessive use of DEET can cause severe toxic reactions in the digestive system, circulatory system, nervous system, and skin in humans and other mammals, as well as metabolic and mental abnormalities, leading to encephalopathy, respiratory depression, seizures, coma, and deep hypotension [23]. When DEET is used at > 50–75% concentrations, it causes serious skin reactions, including mild skin irritation [100] and immune and non-immune contact urticaria [101]. Therefore, direct application of high-concentration DEET formulations to the skin is generally not recommended. DEET can also cause significant irritation to the eyes and mucous membranes, rendering it unsuitable for facial application [24]. Therefore, its use at < 10% concentration is recommended for children aged 2–12 years. Furthermore, pregnant women and infants < 6 months of age should not use insect-repellent products containing DEET [21].

Furthermore, DEET possesses certain physicochemical properties and sensory drawbacks that may affect consumer acceptance. It has a pungent odor and an oily texture, which can lead to an unpleasant sensation when applied to the skin [26]. These characteristics have contributed to consumer reluctance to use it. Moreover, DEET has been shown to reduce the efficacy of sunscreen products; therefore, when both are needed, sunscreen should be applied first, and concurrent use is generally discouraged [87]. In addition, DEET has a corrosive effect and damages materials such as spandex, rayon, acetate fabrics, colored leather, and certain plastics [25]. Besides, relevant studies have shown that DEET is released into waste streams during production and use, contaminating aquatic systems and soil ecosystems [28].

Given the associated risks and limitations of DEET, observing stringent precautions when using DEET-containing products is recommended to ensure user safety and minimize the environmental impact.

#### N, N-diethylphenylacetamide (DEPA)

DEPA (Fig. 6) is a non-toxic, non-stimulating, cost-effective synthetic mosquito repellent with a colorless viscous liquid and mild fragrance. DEPA was synthesized by Kalyanasundaram at the Defense Research and Development Establishment (DRDE) in Gwalior, India [102]. At that time, DEET was expensive, and it was impossible to obtain 3-methybenzoic acid to produce DEET in India. Therefore, based on DEET's skeleton structure, the researchers found a new mosquito repellent, N,N-diethylphenylacetamide [29]. Toxicological evaluations confirmed its safety [103], and DEPA was subsequently adopted by the Indian Armed Forces as a substitute for DEET. It provided effective personal protection against mosquitoes for > 8 h [102].

**Fig. 11:**
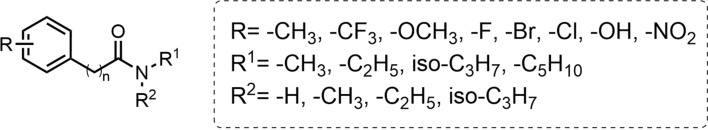
The chemical structure and structure-activity relationship of DEPA

In DEPA's structure, both the benzene ring and the amide group play crucial roles in mosquito-repellent activity (Fig. 11). The mosquito-repellent activity increases with elongation of the carbon chain between the benzene ring and the amide group (with *n* ≤ 4). When the substituent on the benzene ring is methyl or methoxy, the compounds exhibit favorable repellent activity, and the activity of *para*- and *meta*-substituted compounds is superior to that of *ortho*-substituted ones. For the chloro substituent on the benzene ring, *meta*- and *para*-substitutions are beneficial to repellent activity. For fluoro substituent on the benzene ring, *meta*-substitution results in better activity than *ortho*- or *para*-substitution. R^1^ and R^2^ can be substituted by methyl, ethyl, or isopropyl, all of which yield compounds with a high mosquito-repellent activity. Among these, ethyl substitution leads to the most significant activity. When the benzene ring is substituted by methyl, chlorine, or bromine, the mosquito-repellent activity is comparable to that of DEET. Additionally, when the benzene ring is substituted by a trifluoromethyl group, the repellent activity is even better than that of DEET, while other substitutions result in weaker activity than that of DEET. R^1^ and R^2^ can also form a nitrogen-containing five-membered ring with the nitrogen atom, which exhibits some repellent activity [88, 91, 104].

The repellent mechanism of DEPA is currently unknown and may be due to stimulation of mosquitoes' antennal receptors [31]. Regarding its repellent efficacy and safety, Kalyanasundaram et al. [29] reported that DEPA exhibits broad-spectrum repellent activity. Its effective duration exceeds 8 h, meeting the requirement for long-lasting protection. In comparative studies, DEPA demonstrated repellent performance comparable to that of DEET, and superior to that of dimethyl phthalate (commonly known as “mosquito ester”). According to toxicological studies, DEPA is safe for human use [30] and is not cytotoxic or mutational [105], which increases its applicability for direct application to the skin. Moreover, due to its low synthesis cost, DEPA is an economically viable option in developing countries [70].

### Piperidine-based repellents

#### Icaridin

Icaridin (Fig. 6), also known as picaridin, KBR 3023, or Bayrepel™, is a long-acting, broad-spectrum mosquito repellent developed by Bayer in the 1980s using molecular models [70]. Chemically, it is designated as 1-(1-methylpropoxycarbonyl)-2-(2-hydroxyethyl)piperidine, and it is a colorless, odorless, oily liquid [21]. Icaridin was first introduced to the European market in the 1990s and subsequently became available in the US in 2005 [106].

**Fig. 12:**
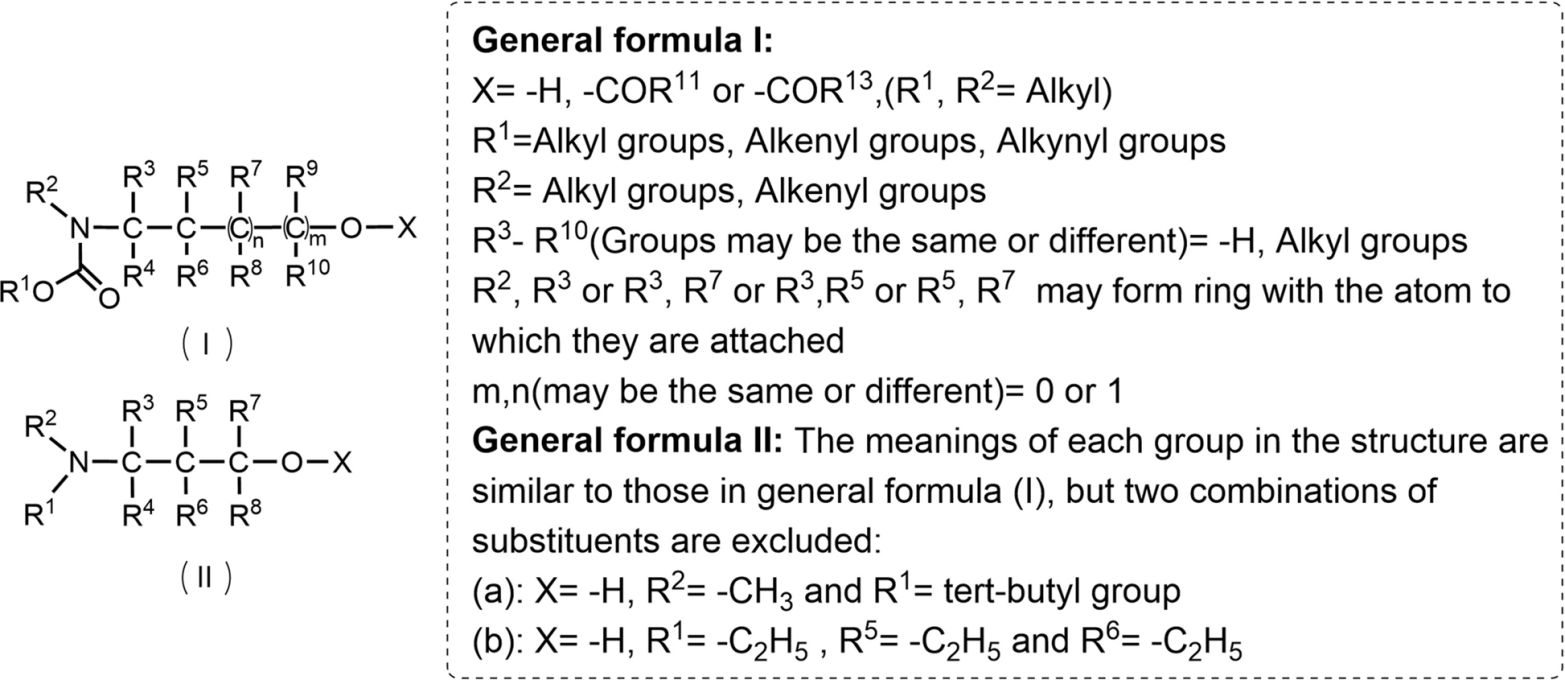
The structure-activity relationship of icaridin

Icaridin is derived from the structural basis of α-ω-aminohydrin (Fig. 12). General formula I exhibits significant mosquito-repellent activity when the substituents meet the following two conditions:

(i) X is hydrogen, COR^11^, or COR^13^ (where R^11^ = R^13^ = C_1_–C_6_ alkyl); R^1^ is C_1_–C_7_ alkyl, C_3_-C_7_ alkenyl, or C_2_-C_7_ alkynyl; R^2^ is C_1_–C_6_ alkyl; R^3^–R^8^ are hydrogen or C_1_–C_6_ alkyl; R^2^ and R^3^ or R^3^ and R^5^ form a five- or six-membered ring; n = 1 and m = 0. (ii) X is hydrogen or COR^13^ (where R^13^ = C_1_–C_6_ alkyl); R^1^ is C_1_–C_7_ alkyl, C_3_–C_7_ alkenyl, or alkynyl; R^4^–R^8^ are hydrogen or C_1_–C_6_ alkyl; R^2^ and R^3^ form a five- or six-membered ring; m = 1 and m = 0 (note: this might contain a typo, possibly intended as “n = 1 and m = 0”) [107].

General formula II shows significant mosquito-repellent activity when the substituents meet the following two conditions:

(i) X is hydrogen, COR^11^ or COR^13^ (where R^11^ = R^13^ = C_1_–C_6_ alkyl); R^1^ is C_1_–C_7_ alkyl, C_3_–C_7_ alkenyl, or C_2_–C_7_ alkynyl; R^2^ is C_1_–C_6_ alkyl; R^3^–R^8^ are hydrogen or C_1_–C_6_ alkyl; R^2^ and R^3^ or R^3^ and R^5^ form a five- or six-membered ring. (ii) X is hydrogen or COR^13^ (where R^13^ = C_1_–C_4_ alkyl); R^1^ is C_3_–C_4_ alkyl or alkenyl; R^4^–R^8^ are hydrogen; R^2^ and R^3^ form a six-membered ring [107].

In General Formula III, variations in the R^1^ group significantly impact activity: substitution with –C_4_H_9_ or –OC_4_H_9_ confers mosquito-repellent activity, with the latter (–OC_4_H_9_) exhibiting optimal activity, while –NC_4_H_9_ substitution results in no activity. When R^2^ is substituted with –CH_2_CH_2_OH, the position of the substituent greatly affects activity: the 2-position substitution yields the best mosquito-repellent activity, whereas substitutions at the 3- or 4-position result in no activity [108].

Icaridin's precise mechanism of action remains largely unclear; however, studies suggest that it functions via a mechanism similar to activating the odorant receptor CquiOR136 in *Culex quinquefasciatus* [109]. Icaridin is thought to act primarily by forming a vapor barrier that prevents mosquitoes from biting and interferes with the function of olfactory sensory neurons, thereby disrupting host detection [32, 110].

Icaridin is commonly formulated as a spray or lotion at concentrations of 15–20%, and multiple studies have demonstrated that its repellent efficacy is comparable to that of DEET [32, 111, 112]. Icaridin is widely used in Europe at a concentration of 20%, providing effective protection for up to 8–10 h [32]. It evaporates more slowly from the skin surface than DEET, which may contribute to its longer-lasting efficacy, particularly at higher concentrations [21]. In a field study conducted in Burkina Faso, icaridin demonstrated a higher protection rate against multiple *Anopheles* species than DEET, indicating superior field efficacy under certain conditions [113]. Due to its favorable safety profile, efficacy, and cosmetic acceptability, icaridin is recommended by the World Health Organization as a mosquito repellent for malaria prevention [32].

Unlike DEET, icaridin exhibits several advantageous repellent properties. It is colorless, odorless, and generally better accepted by users because of its mild olfactory profile. Icaridin offers a more comfortable and refreshing application experience, being non-greasy and non-sticky. Moreover, it is biodegradable and does not interact adversely with synthetic materials, such as plastics [32, 33]. In contrast, DEET is known to exert a significant plasticizing effect, which can damage various polymer-based materials. In addition, the compound has low toxicity. The EPA [114] states that icaridin has no obvious toxicological effects in animal and human experiments, its toxicity-induced hallucinations are rare and easily reversed under supportive therapy, and normal use does not cause health problems. Icaridin is also less irritating to the skin and to smell [32], has no serious adverse effects on the nervous system, and increases user compliance [33, 114]. Johan et al. [115] conducted studies in Cambodia and found that adverse reactions and misuse of icaridin were infrequent and typically mild, supporting its safety for use in malaria control programs. However, studies have indicated that icaridin may cause eye and skin irritation in sensitive individuals [34] and may also discolor materials and animal leather clothing [24]. Additionally, there have been reports of nonspecific adverse effects, such as hypersensitivity reactions and reversible loss of consciousness. Nonetheless, these symptoms are generally mild and tend to resolve either spontaneously or with appropriate medical treatment [23]. In conclusion, icaridin is less toxic and better tolerated than DEET, and formulations containing 5–10% icaridin are considered safe for use in children > 6 months old [34]; it can be used as a substitute for DEET [21]. However, although icaridin is widely used in Europe and Australia, it was only introduced to the Chinese market in 2012 and remains relatively expensive. Therefore, commercially available icaridin-based repellents are still limited on the Chinese market [35].

#### SS220

SS220 (Fig. 6), chemically known as (1S, 2S)-2-methylpiperidine-3-cyclohexene-1-carboxamide, is a synthetic mosquito repellent derived from the compound mixture AI3-37,220 (Fig. 6), which was first synthesized by the US Department of Agriculture in 1978 [70, 116]. The compound was later designated SS220 and patented in 2003 [36].

**Fig. 13:**
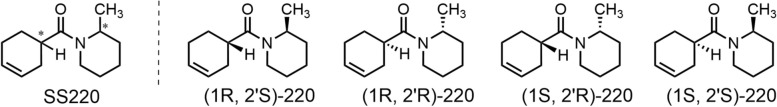
The chemical structures of four SS220 stereoisomers

SS220 contains two asymmetric centers, allowing for the formation of four stereoisomers (Fig. 13): (1R, 2’R), (1S, 2’S), (1R, 2’S), and (1S, 2’R). However, not all isomers have good repellent activity, and the stereoisomers (1S, 2S) and (1R, 2S) are 2.8–3.1 and 1.6 and 1.6–1.8 times better than those of other stereoisomers, respectively. Notably, (1S, 2S) stereoisomers were 2.5 times more effective than *Ae. aegypti* at the same doses [116].

According to Klun et al. [36, 117], SS220 was effective against *Anopheles* mosquito and *Ae. aegypti* and was better against *Ae. aegypti* than the homopiperidine derivative KBR 3023 (icaridin). However, its repellent performance was inferior to that of diethylphenylacetamide (DEPA) (Fig. 6) [118]. According to a report by the US Department of Agriculture, SS220 exhibits repellent efficacy comparable to that of a 33% DEET formulation. However, rigorous field evaluations of its performance remain limited [36]. Toxicological and pharmacological assessments have demonstrated that SS220 is characterized by low toxicity and minimal irritation, and it does not pose significant health risks to humans [119–122].

SS220 exhibits low volatility and reduced dermal absorption potential, contributing to a superior safety profile to that of DEET [70]. In addition, SS220 has a mild, fruity scent, a non-oily consistency, and a pleasant skin feel, which enhances user acceptability. Unlike DEET, SS220 does not degrade plastics and exhibits negligible plasticizing effects [123], making it more compatible with a broader range of materials. However, the disadvantage of SS220 is that it is a single stereoisomer that requires separation from a racemic mixture, so the production cost is higher than that of other racemic synthetic repellents [70]. In addition, SS220 has not been registered because of its high development cost [70], making it difficult to compete with DEET in the repellent market.

### Esters

#### Ethyl butylacetylaminopropionate (IR3535)

IR3535 (Fig. 6), chemically known as ethyl butylacetylaminopropionate and commercially registered under the trade name “Immerning,” is a broad-spectrum and highly effective synthetic mosquito repellent. It was developed by Merck in 1975, based on the chemical structure of the natural amino acid alanine [124]. IR3535 is an odorless, transparent, oily liquid. It is volatile at atmospheric pressure and room temperature and slightly soluble in water, but soluble in organic solvents [125]. Following its approval by the US Food and Drug Administration (FDA) in 1999, IR3535 was introduced to the US market not only as an insect repellent but also for its emollient and moisturizing properties. It has been promoted as an ideal alternative to DEET due to its lower toxicity and reduced skin irritation[21].

**Fig. 14:**
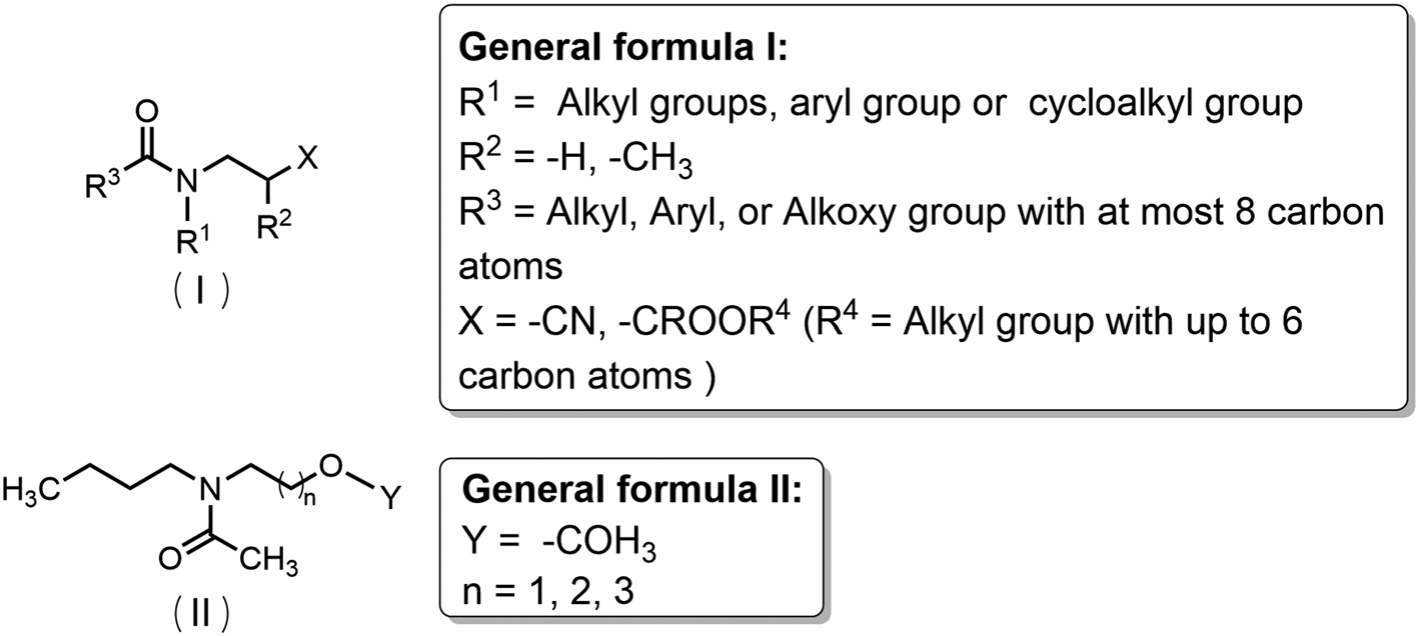
The structure-activity relationship of IR3535

IR3535 is chemically derived from the structural framework of alanine, a naturally occurring amino acid, with the alkylamino group functioning as an essential moiety for its repellent activity. In General Formula I (Fig. 14), repellent activity is observed when R^1^ is substituted with C_2_–C_4_ alkyl groups; substitutions of R^2^ with H or -CH_3_ exert minimal influence on repellent activity, although H-substituted R^2^ yields the highest efficacy. Repellent activity is also conferred when R^3^ is substituted with C_1_-C_4_ alkyl groups. However, when the total number of carbon atoms in the substituent of R^1^ and R^3^ is ≥ 6—particularly when these substituents are cycloaliphatic or aromatic—repellent activity decreases significantly or even becomes ineffective. Additionally, substitutions of X with -CN or -C(O)OR^4^ (where R^4^ ranges from methyl to n-butyl) result in repellent activity, with R^4^ as methyl to n-butyl delivering the best repellent efficacy [124]. In General Formula II (Fig. 14), mosquito-repellent activity is observed when Y is substituted with -OC_2_H_5_. Specifically, the activity is relatively weak when n = 1, improves when n = 2, and reaches the optimal level when n = 3 [108].

The mechanism of action of IR3535 involves odor-dependent inhibition of olfactory neurons and is associated with odor-independent excitatory activity [110]. Related studies have shown that, like DEET, IR3535 may target one or several odor receptors in mosquitoes, depending on the presence or absence of another odor molecule. It is hypothesized that IR3535 forms a vapor-phase barrier that interferes with mosquito host-seeking behavior, thereby preventing contact with human skin [109].

IR3535 is chemically stable under use conditions, with high thermal stability [126] and long duration of action, and it is most effective against *Culex* species. A 20% IR3535 formulation provides 7–10 h of complete protection against *Culex* and *Aedes* mosquitoes and approximately 3–4 h against *Anopheles* species [37]. In a comparative study, Thavara et al. [127] evaluated the repellent efficacy of 20% IR3535 and DEET against multiple mosquito species, including *Anopheles dirus* and *Culex quinquefasciatus*, and found that the protection time provided by IR3535 was generally shorter than that of DEET across all tested species. Although IR3535 is generally less effective than DEET in terms of repellent duration, it offers superior biocompatibility and lower toxicity. Extensive toxicological studies have confirmed its safety profile, supporting its use in the development of low-toxicity topical repellent formulations suitable for children > 6 months old and pregnant women [38]. There were no reported adverse effects or developmental toxicity when it was initially sold as a skin emollient and moisturizer [24], but animal studies found that pure mosquito repellent was highly toxic, with acute skin and digestive tract reactions, and chronic toxicity manifested as a body mass change, gastric mucous damage, and reproductive toxicity [23].

Pure IR3535 has an oily consistency, and the sensory experience upon application is not pleasant. When applied to the skin, it tends to feel sticky and greasy. Additionally, due to its low molecular weight, IR3535 can penetrate the skin, potentially causing localized irritation, particularly to sensitive areas such as the eyes [21]. Therefore, it is not recommended to smear pure IR3535 directly on the skin or face. Like DEET, IR3535 may dissolve or destroy synthetic materials such as clothing and plastics [38]. At present, there are many mosquito-repellent products containing IR3535, including aerosols, emulsions, pump sprays, and wipes, with a concentration of 7.5–19.7% [4]. Compared with DEET, it is less toxic and less irritating. Therefore, it is still a powerful substitute for DEET in endemic areas.

#### DMP

DMP (Fig. 6), also known as dimethyl 1,2-phthalate, is one of the first chemical synthetic mosquito repellents [70]. Initially developed as an industrial solvent rather than a repellent, DMP was often employed as a carrier to test solid repellent compounds [70]. For instance, certain repellents provided only 45 min of protection in the presence of DMP compared to up to 7 h in its absence [39]. However, DMP itself was later found to possess intrinsic repellent properties. Therefore, it was widely used as a mosquito repellent between the 1940s and 1980s [31].

**Fig. 15:**
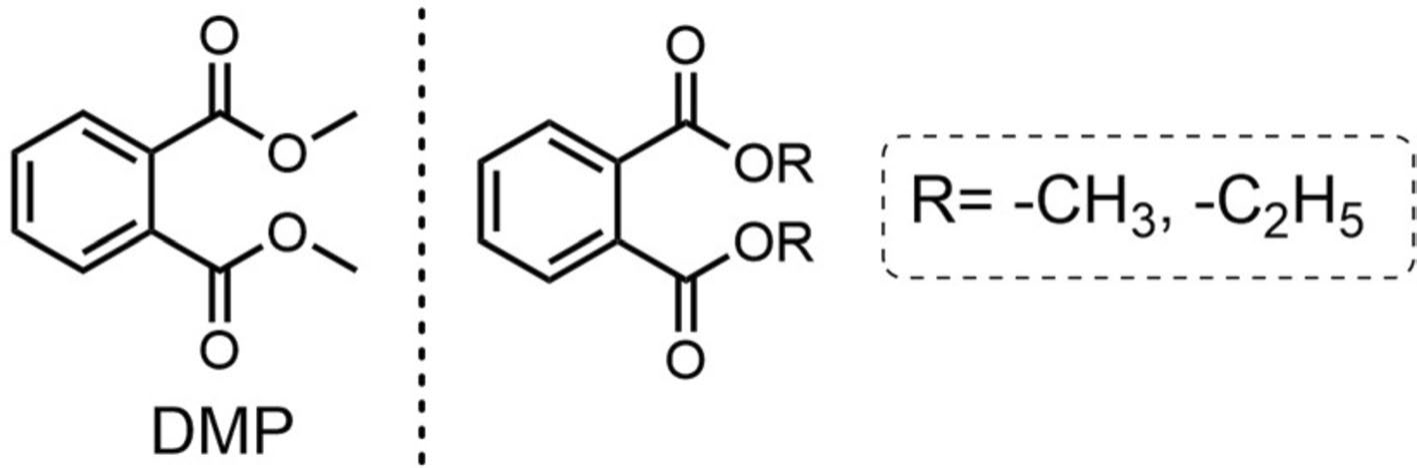
The chemical structure and structure-activity relationship of DMP

DMP comprises a benzene ring and a carboxylate, which endows it with certain mosquito-repellent activity (Fig. 15). In its structural general formula, R can be substituted by methyl or ethyl groups: methyl substitution confers favorable mosquito-repellent activity, while ethyl substitution results in no activity [108].

During World War II, existing mosquito repellents were insufficient to provide adequate protection for military personnel deployed overseas. Therefore, a new formulation—comprising six parts dimethyl phthalate (DMP), two parts indalone (Fig. 6), and two parts ethylhexanediol—was developed and designated “6-2-2” or M-250. Following this, DMP was also used as a repellent against *Aedes* mosquitoes. However, it was soon replaced by more effective active ingredients, including para-menthane-3,8-diol (PMD), DEET, DEPA, and other novel repellent compounds [70]. Experimental studies have shown that the repellent efficacy of dimethyl phthalate (DMP) is generally lower than that of DEET, and its effectiveness varies significantly across different mosquito species [128]. Additionally, the minimum effective dose required to prevent mosquito bites is considerably higher for DMP than for DEET. Specifically, DMP requires approximately 8–8.15 mg per square inch, whereas DEET achieves comparable protection at a much lower dose of 0.36–0.50 mg per square inch [129].

Adverse skin reactions after topical application of dimethyl phthalate have not been reported [40]. Relevant toxicological studies indicated that DMP had low toxicity (LD_50_: 6900 mg/kg) in mice and was not toxic to rabbits exposed to 1000 mg/kg per day [31]. Despite these findings, DMP has been withdrawn from the market as a mosquito repellent because of concerns about its limited efficacy and potential toxicity [128]. Nevertheless, owing to its favorable properties such as film-forming ability, adhesion, and water resistance, DMP is currently used in the pharmaceutical industry primarily as a solvent [41].

## Active ingredients and mechanism of action in natural mosquito repellents

**Fig. 16:**
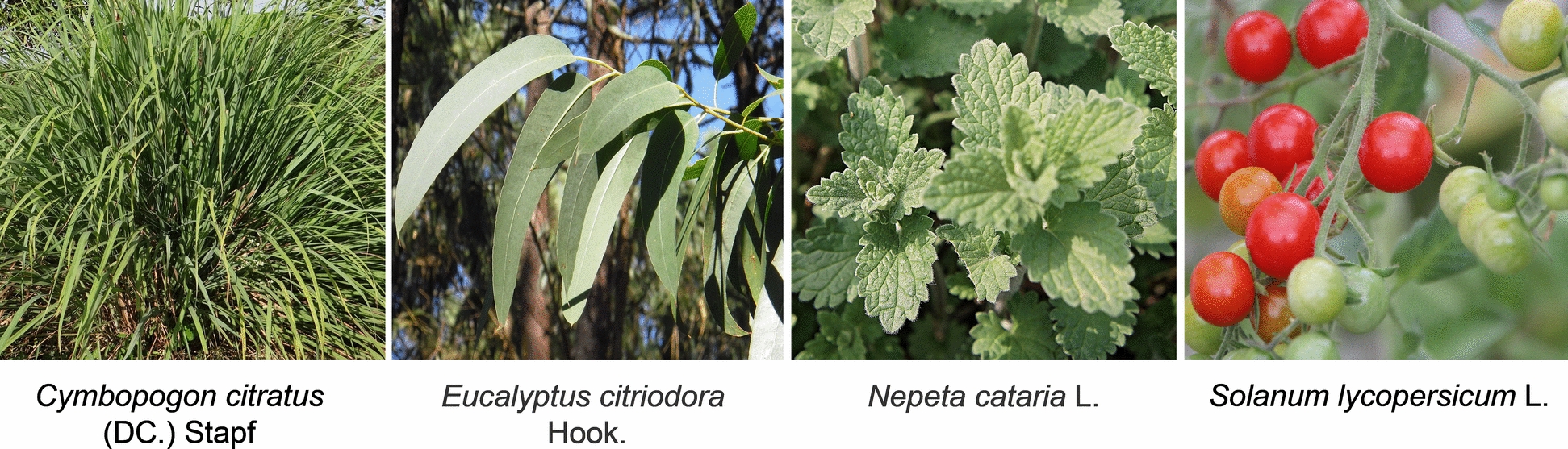
Natural mosquito-repelling plants. Images were taken from Pixabay (https://pixabay.com)

Natural mosquito repellents are typically derived from plant extracts (Fig. 16). They are readily available, biodegradable, leave minimal residues, and offer significant environmental benefits, making them highly suitable for widespread application. Their core repellent efficacy stems from active components within plant secondary metabolites, which can be categorized into terpenes, aldehydes, esters, alcohols, and long-chain fatty ketones. This section will focus on representative essential oil active ingredients, the mechanisms of action involving essential oil-related receptors, and the principles of visual effects.

**Fig. 17:**
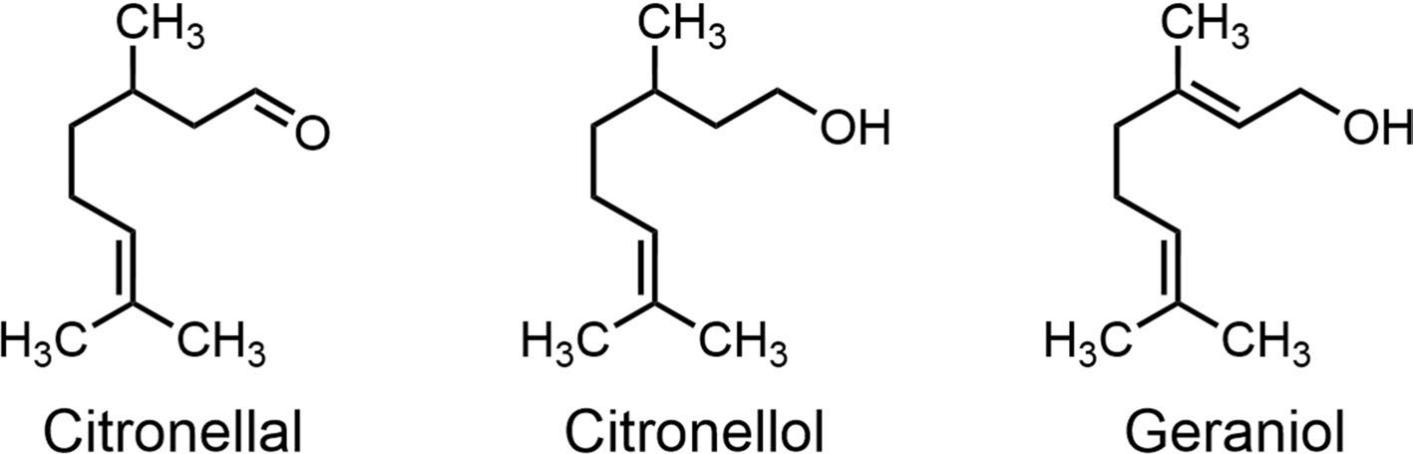
The chemical structure of citronellal

Citronella oil (Fig. 17), also known as lemongrass oil or “male grass” oil, is a volatile essential oil extracted from *Cymbopogon nardus*. It contains multiple components, including citronellal, citronellol, and geraniol. Citronellal is widely recognized as the primary active ingredient responsible for its mosquito-repellent properties, accounting for 60%–80% of its composition [33]. It exhibits particularly potent repellent effects against mosquitoes of the *Anopheles* and *Culex* genera [23]. However, some commercial mosquito repellents containing ≤ 12% citronella oil demonstrate significantly shorter protection durations than synthetic repellents like IR3535 [98].

**Fig. 18:**
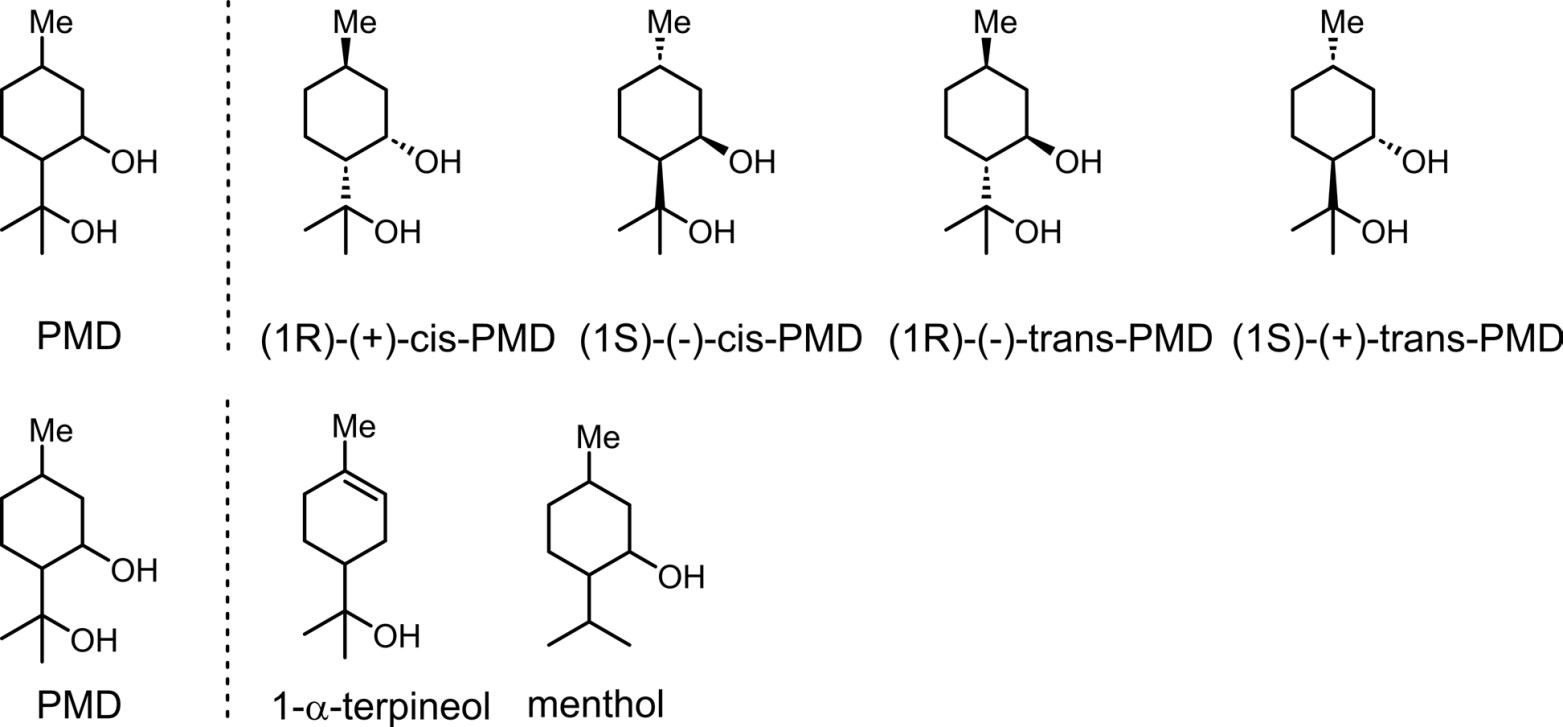
The chemical structures of four stereoisomers of PMD and the chemical structures of analogs of PMD

Lemon eucalyptus oil is a plant essential oil extracted from lemon eucalyptus trees [33], with the primary active ingredient being p-menthane-3,8-diol (PMD) (Fig. 18) [33]. As a monoterpene compound, it exhibits low volatility [130], a physicochemical property enabling it to provide approximately 5 h of mosquito protection [131]. Formulations containing 20% PMD demonstrated superior efficacy to 10% DEET within 8 h, while compared to 30% DEET, it performs slightly less effectively [131]. At a 15% concentration, PMD provides approximately 4.4 h of protection [132]. PMD exists in four stereoisomeric forms (Fig. 18). Repellent efficacy studies indicate all four isomers exhibit comparable repellent activity against *Anopheles gambit*. Structural analogs of PMD (Fig. 18), such as 1-α-terpineol (hydroxide at the C-8 position with an unsaturated structure) and menthol (hydroxide at the C-3 position), exhibit no mosquito-repellent activity. This indicates that subtle differences in hydroxide position and molecular saturation significantly influence biological activity [133].

**Fig. 19:**
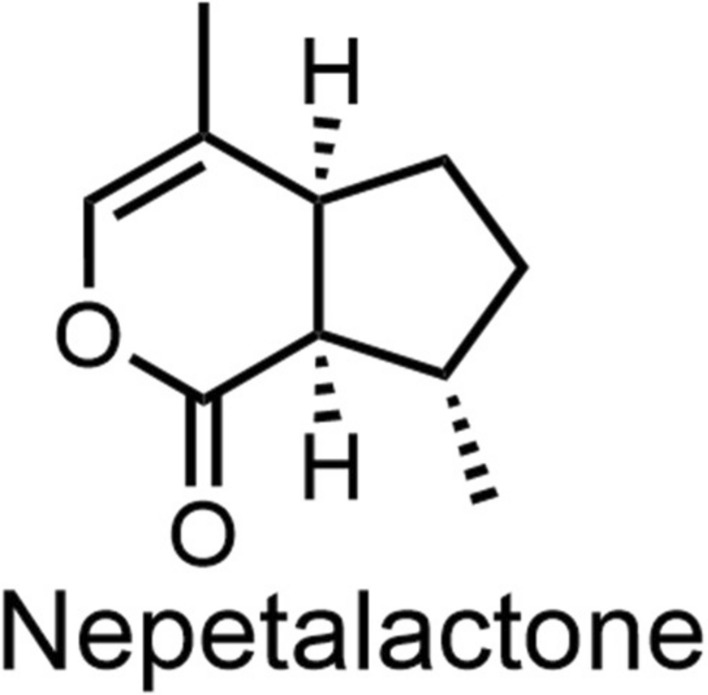
The chemical structures of analogs of nepetalactone

Catnip oil, a representative ester -based essential oil with a distinctive cooling aroma, is native to temperate and tropical regions of Asia and Europe [134]. It is composed of multiple constituents, with the primary mosquito-repellent component being nepetalactone (Fig. 19)—a terpenoid lactone consisting of two isoprene units [135, 136]. Junwei Zhu et al. [137] evaluated the topical repellent activity of five plant essential oils against *Aedes albopictus*. Among the tested oils, catnip oil exhibited the strongest repellent activity, with an optimal effective concentration of 23 mg/ml.

**Fig. 20:**
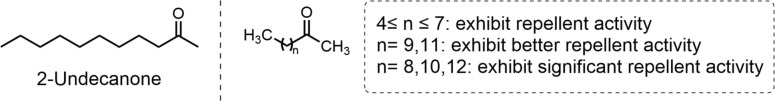
The structure-activity relationship of BioUD

BioUD, a representative long-chain aliphatic ketone, is a novel botanical repellent registered with the US Environmental Protection Agency (EPA) in April 2007 [138]. Its active ingredient, 2-undecanone (Fig. 20), is a natural compound isolated from glandular trichomes on the stems and leaves of wild tomato (*Solanum* spp.) [139]. Among newly developed botanical repellents, BioUD exhibits potent repellency comparable to 20% DEET and superior to IR3535 under certain conditions [106]. Roe et al. [138] found that 25% and 30% BioUD concentrations outperformed DEET, whereas its efficacy at 15% was comparatively lower. In a related study, Innocent et al. [140] evaluated the repellent activity of a homologous series of long-chain aliphatic methyl ketones ranging from C7 to C15 (Fig. 20). The results indicated that chain length significantly influenced repellency, with compounds containing 11 to 15 carbon atoms exhibiting stronger activity than shorter-chain analogs (C7 to C10). Furthermore, within the C11-C15 carbon atom range, aliphatic methyl ketone series with odd-numbered carbon chains (e.g. 2-undecanone, 2-tridecanone, 2-pentadecanone) exhibited significantly greater repellent efficacy than their even-numbered counterparts (e.g. 2-decane-2-one, 2-dodecanone), a trend particularly evident at higher concentrations.

**Fig. 21:**
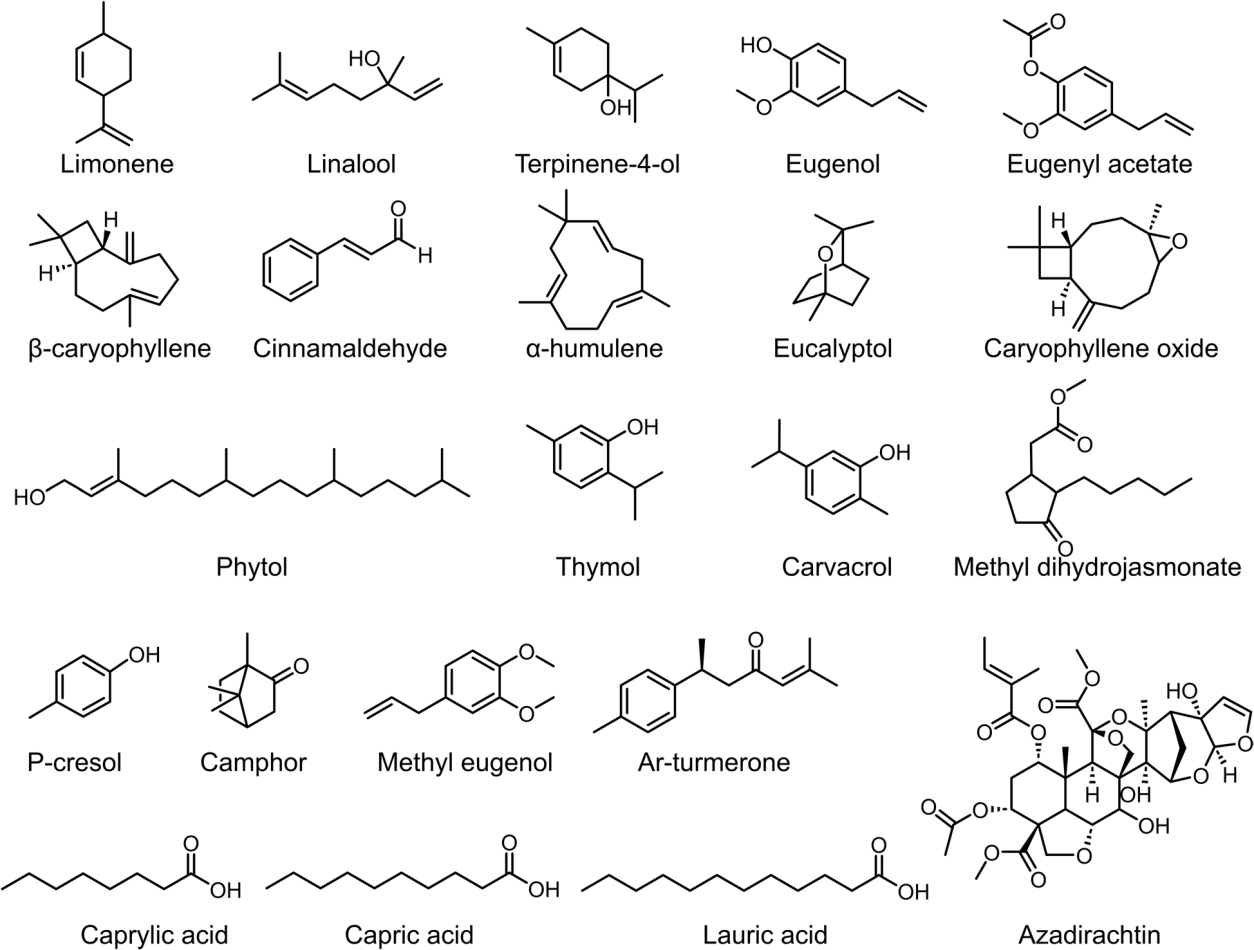
Structure of active components in plant-based essential oil

Orange peel essential oil is extracted from the peels of citrus fruits (e.g. *Citrus macroptera*), and its primary bioactive component is limonene (86.76%) (Fig. 21). It provides mosquito repellent protection lasting 5.16 ± 0.23 h. When blended with vanilla essential oil at a 1:1 ratio, the protective duration extends to 6.33 h [141]. A 20% concentration of orange peel essential oil extract repels mosquitoes for 2 h, while a 25% concentration extends the repellent to 5 h. Only minor side effects were observed, including transient skin itching and occasional sneezing [142].

*Homalomena aromatica* oil is a traditional mosquito repellent used by residents in Northeast India [143], primarily composed of linalool (52.35%), terpinene-4-ol (18.18%), and trace amounts of limonene (Fig. 21). Its repellent protection lasts up to 6.16 ± 0.23 h, although it is less potent than DEET [141].

Clove essential oil is a natural plant essential oil extracted from the clove plant (*Syzygium aromaticum*), with its primary active constituents being eugenol (76.2%), eugenyl acetate (17.8%), and β-caryophyllene (4.1%) (Fig. 21) [144]. Clove oil exhibits significant repellent activity against *Culex pipiens pallens*, achieving repellent efficacy > 80% at a concentration of 0.005 mg/cm^2^, with efficacy surpassing that of commercial citronella oil [145].

Cinnamon essential oil is extracted from the bark of *Cinnamomum verum* J. Presl, with cinnamaldehyde (Fig. 21) constituting 86.1% of its volatile constituents—the core component responsible for its mosquito-repellent activity [146]. At a 10% concentration, cinnamon essential oil achieves an 83.8% repellent efficacy, with an 82% repellent efficacy against *Aedes albopictus*. At a concentration of 0.153 mg/cm^2^, trans-cinnamaldehyde provides 93% protection against *Aedes aegypti* after 40 min of exposure, and at 0.051 mg/cm^2^, it provides 87% protection after 30 min, with efficacy approaching that of the conventional repellent DEET [147, 148].

Artemisia essential oil is a volatile aromatic substance extracted from the leaves and stems of mugwort. Its primary active constituents include α-humulene, eucalyptol, β-caryophyllene, chlorophyll, and caryophyllene oxide (Fig. 21) [149, 150]. This essential oil at a concentration of 500 μg/ml exhibits significant larvicidal efficacy against both third- and fourth-instar larvae of *Aedes aegypti* mosquitoes. Furthermore, at a 50% concentration, the oil provides repellent activity against female *Aedes aegypti* mosquitoes for up to 60 min[149].

Thyme essential oil contains monoterpenes as its primary active constituents, with key components including thymol and carvacrol (Fig. 21) [151]. Its mosquito-repellent protection lasts 1.5–3.5 h, and the duration of this protective effect varies with concentration [152]. In experimental applications, when thyme essential oil was administered at a 0.05% concentration on hairless mice, it achieved 91% protection efficacy and significantly prolonged the intervals between mosquito bites [151]. However, concentrations > 25% lead to an unpleasant odor and may cause skin irritation, limiting its practical use at high doses [152].

Jasmine essential oil is a volatile aromatic oil extracted from the flowers of plants belonging to the genus *Jasminum* within the Oleaceae family (such as *Jasminum sambac* and *Jasminum grandiflorum*). Its primary active constituents are methyl dihydrojasmonate and p-cresol (Fig. 21), both of which achieved a 100% repellent efficacy against *Aedes albopictus*. A repellent solution formulated with a 15:1 ratio provides effective protection for up to 6.25 h [153]. Furthermore, perfume products containing dihydrojasmonate methyl ester exhibit mosquito-repellent activity lasting up to 6 h [154].

Neem oil is a natural oil extracted from the seeds of the neem tree (*Azadirachta indica* A. Juss.), belonging to the Meliaceae family. Its active component, azadirachtin (Fig. 21), possesses insecticidal and repellent properties [155]. Neem oil has been demonstrated to repel multiple mosquito species, particularly those belonging to the genus *Anopheles*. For instance, neem seed oil creams at 17.5% and 20% concentrations achieve an 87.5% repellent rate against *Anopheles* mosquitoes [156]. Field trials indicate that a 20% neem oil solution achieves an average repellent efficacy of 71%, providing protection for up to 3 h [157].

Basil essential oil is a volatile aromatic oil extracted from the leaves and inflorescences of plants belonging to the genus *Ocimum* within the Lamiaceae family. Its active constituents include camphor, caryophyllene oxide, eucalyptol, and methyl eugenol (Fig. 21) [158]. This essential oil provides mosquito repellent activity lasting 90–120 min, with clove basil oil exhibiting repellent effects exceeding 2 h and sweet basil oil demonstrating the strongest larvicidal activity [158, 159].

Coconut oil typically refers to the fat extracted from the endosperm of the coconut, a plant belonging to the genus *Cocos* within the palm family. Its mosquito-repellent active components are medium-chain fatty acids C8–C12 (caprylic acid, capric acid, lauric acid) (Fig. 21). These components exhibit concentration-dependent repellent efficacy against mosquitoes, demonstrating significant repellent efficacy when applied topically at high concentrations. Against *Aedes aegypti* mosquitoes, the repellent rate exceeds 90%, whereas DEET achieves only approximately 50% repellent efficacy against similar blood-feeding insects [160]. Additionally, formulations derived from these components form a protective barrier on skin or animal surfaces, enabling the gradual release of active ingredients. This significantly extends repellent efficacy compared to the volatile DEET [160].

Turmeric essential oil primarily contains ar-turmerone (Fig. 21), which exhibits repellent and insecticidal activity. At 138.86 ppm, curcumene causes 50% mortality in *Culex pipiens* larvae [161]. A 25% concentration of turmeric extract provides up to 3.5 h of mosquito-repellent protection for humans, offering defense against multiple species including *Culex tritaeniorhynchus* and *Anopheles sinensis* while exhibiting no irritation upon application to human skin [162].

### Mechanisms of action

**Fig. 22:**
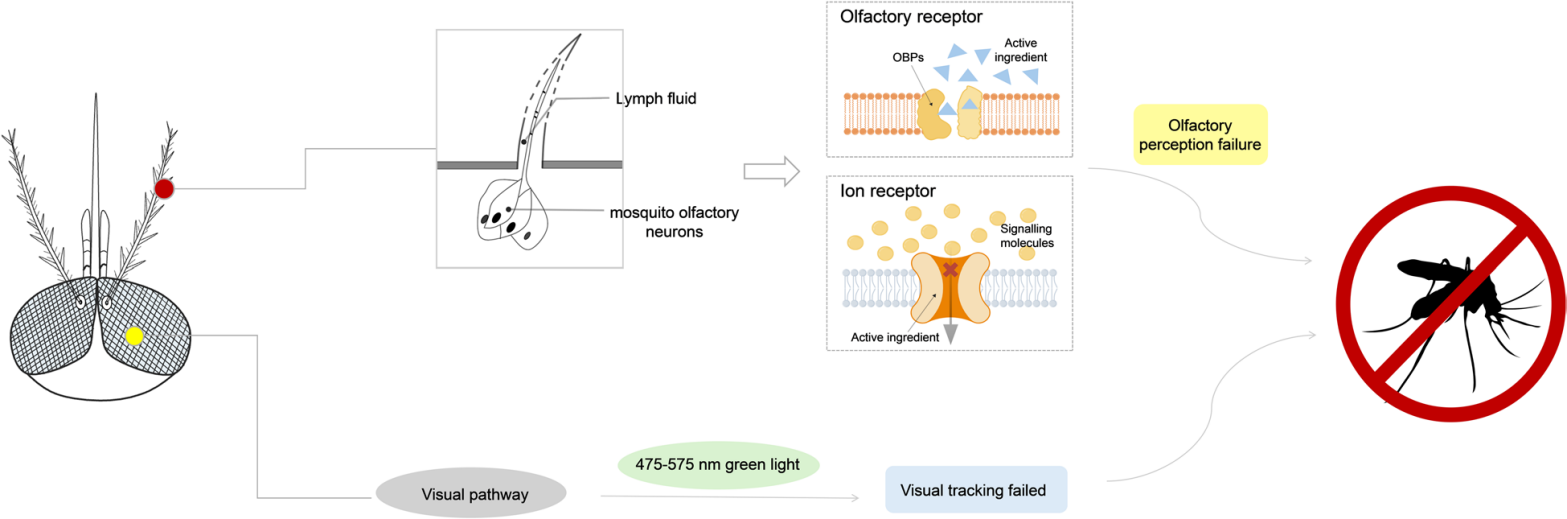
Proposed mechanism of action of natural repellents. Natural mosquito repellents primarily function by disrupting the mosquito’s olfactory system, impairing its ability to detect and recognize host-emitted odor cues. In addition, some active compounds modulate ionotropic receptors, altering neuronal membrane ion permeability and interfering with signal transduction in olfactory neurons. Synergistic interactions among multiple constituents may enhance this interference. At the visual level, essential oil droplets can scatter green light wavelengths to which mosquitoes are highly sensitive, while certain compounds reduce the responsiveness of compound eyes to motion stimuli. Together, these effects disrupt the integration of olfactory and visual cues, ultimately impairing the mosquito’s host-seeking behavior. Images were created with BioGDP.com[20]

The physiological processes triggered by plant extracts remain understudied (Fig. 22). The core mechanism by which active ingredients in natural mosquito repellents exert their effects primarily involves disrupting the mosquito’s olfactory system, impairing its perception and recognition of host odor signals, thereby preventing the mosquito from locating its host. Concurrently, certain components also act upon the mosquito’s ion receptors, altering the ion permeability of nerve cell membranes to interfere with the transmission of olfactory signals. Furthermore, synergistic interactions may occur between different components, collectively enhancing the disruption of the mosquito’s olfactory system.

Certain active ingredients can bind directly to olfactory receptors on mosquito antennae, disrupting normal receptor-mediated signal transduction by either activating or inhibiting these receptors[163]. For example, 2-undecanone (2-U) can directly bind to the olfactory receptors of *Aedes aegypti* (AaOR2, AaOR8), exerting bidirectional regulatory effects. In the absence of odor stimuli, the active compound selectively activates specific olfactory receptors. Conversely, when odor molecules of host origin are present, it potently inhibits their normal responses of the receptor. This dual functionality directly activates olfactory sensory neurons (OSNs) and interferes with signal transduction pathways [110]. PMD exhibits partial inhibitory effects on the AaOR2 and AaOR8 receptors in *Aedes aegypti* [164] while simultaneously activating the CquiOR136 and CquiOR32 receptors in *Culex quinquefasciatus* in the absence of agonists [109, 165]. The core component of jasmine essential oil, methyl dihydrojasmonate, activates the DEET-sensitive olfactory receptor CquiOR136 in *Anopheles sinensis*, disrupting olfactory signal transduction and preventing mosquitoes from locating hosts [166]. The primary function of olfactory binding proteins is to transport hydrophobic odor molecules via antennal lymph to olfactory receptors, constituting the initial stage of odor recognition in mosquitoes [167]. Certain active components in natural repellents selectively bind to olfactory binding proteins, impairing their transport function for odor molecules and thereby disrupting mosquito olfactory recognition. For instance, PMD exhibits selective binding affinity for different olfactory binding proteins, regulating mosquito olfaction through multiple pathways by simultaneously binding to olfactory binding proteins while inhibiting or activating specific odor receptors [168]. Although limonene exhibits low binding affinity for the Gambian mosquito olfactory binding protein AgamOBP1, it synergistic effect with components such as linalool in vanilla essential oil enhances disruption of the mosquito olfactory system [141]. Linalool exhibits a binding affinity of  − 57.787 kcal/mol with AgamOBP1, effectively disrupting the mosquito’s olfactory system for detecting human odor [141]. Cinnamaldehyde exhibits a binding energy below − 5 kcal/mol with the odor-binding protein OBP1 of *Aedes albopictus*, forming a tight complex that interferes with the protein’s recognition and transmission of human volatile signals, thereby disrupting the mosquito’s olfactory homing system [169].

Certain active ingredients target insect ion receptors, altering the ion permeability of nerve cell membranes and disrupting membrane potential, thereby blocking the transmission of olfactory signals [135]. For instance, nepetalactone in catnip oil targets the transient receptor potential channel TRPA1 within mosquitoes, which is closely associated with olfactory signal transduction. This action promotes channel opening, alters ion permeability in the nerve cell membrane, and directly inhibits the transmission of olfactory signals to the central nervous system, significantly diminishing the mosquito’s perception of host odor [170].

Beyond the aforementioned target-specific mechanisms, certain components within plant essential oils exhibit distinct modes of action. Taking turmeric extract as an example, its mechanism varies with concentration. At low concentrations (1.0%), aromatic ar-turmerone predominates, primarily inducing rapid flight in *Aedes aegypti* mosquitoes through contact irritation. At higher concentrations (5.0%), a non-contact repellent effect occurs solely via olfactory signals without physical contact. This concentration-dependent mechanism constitutes a distinctive characteristic of turmeric extract [171].

Additionally, essential oil repellents also exert effects through visual pathways, thereby expanding their functional spectrum (Fig. 22). Mosquitoes exhibit specific spectral preferences—particularly a heightened sensitivity to green light within the 475–575-nm range, which are significantly modulated by olfactory cues. The presence of plant-derived volatiles has been shown to alter mosquito responses to specific light wavelengths by modulating olfactory-visual integration mechanisms [172, 173]. Experimental data show that rosemary oil achieves a repellency rate of 94% against *Aedes aegypti* mosquitoes, while lavender oil achieves 89%, both approaching the repellent efficacy of the chemical repellent DEET. Although the study did not directly observe visual mechanisms, the repellent efficiency significantly exceeded expectations based on olfactory interference alone. This suggests that the volatile oil may form microscopic droplets that scatter blue-green light sensitive to mosquitoes, altering the distribution of ambient light intensity. This optical interference may disrupt the mosquito’s visual system and act in concert with olfactory regulation, producing a behavioral repellent effect [174]. Relevant studies indicate that components such as linalool and α-pinene, lavender essential oil, can “comprehensively disrupt mosquito sensory systems.” While olfactory suppression is the primary recognized mode of action, the use of terms like “omnidirectional sensory interference” suggests involvement of non-olfactory pathways, including visual disruption [151, 175]. For instance, citronellal has been reported to interfere with olfactory signaling while simultaneously reducing the sensitivity of compound eye photoreceptors to moving stimuli. By obstructing the integration of visual-olfactory signals, such compounds further enhance repellent effect [176, 177].

## New formulation development of repellent

**Fig. 23:**
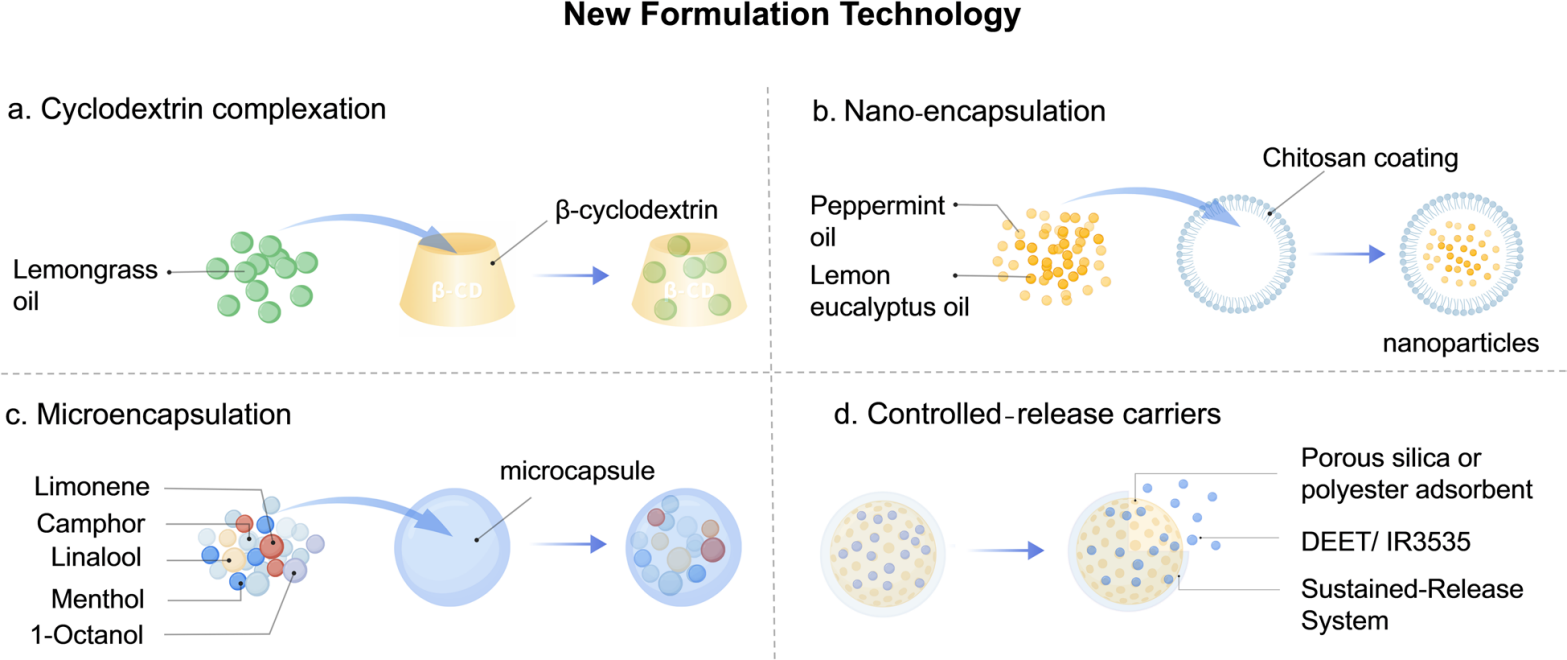
New formulation technology. **a** Cyclodextrin complexation. **b** Nano-encapsulation. **c** Microencapsulation. **d** Controlled-release carriers. Images were created with BioGDP.com[20]

Current mosquito-repellent products, such as lotions, roll-ons, and sprays, exhibited long-term efficacy and typically require frequent reapplication or replacement. To address these limitations, recent research has focused on advanced technologies like cyclodextrin complexation (Fig. 23a), nano-encapsulation (Fig. 23b), microencapsulation (Fig. 23c), and controlled-release carriers (Fig. 23d). Simultaneously, formulation recipes are continually optimized to align with consumer preferences while enhancing sustained release and retention of repellent activity.

At the level of new technologies, Jiang Xuefeng et al. [178] optimized the β-cyclodextrin (β-CD) inclusion process, resulting in a reduction of the essential oil volatilization rate to 36% after 35 days of storage in the β-CD inclusion group. In a separate study, β-CD-complexed citronella oil retained 82% of its active components after 6 months under accelerated light exposure conditions at 40 °C, compared to only 28% in the non-complexed group. Moreover, the inclusion process reduced the overall volatilization rate by 58% [179]. Nanotechnology-based approaches such as nano-emulsification and nano-milling with encapsulation have been employed to achieve controlled release of active ingredients. A study employed a two-step method utilizing chitosan coating to prepare nanoparticles measuring 80–150 nm from peppermint oil and lemon eucalyptus oil [180]. Mosquito-repellent sprays containing these nanoparticles provided up to 10 h of protection, compared to only 3 h for conventional formulations. Under simulated perspiration conditions, the encapsulated group exhibited an active ingredient loss rate of only 8% within 2 h, whereas the non-encapsulated group experienced a loss of 45%. Maitra et al. [181] successfully developed a mosquito-repellent formulation containing essential oils such as limonene, camphor, and linalool using microencapsulation technology. When tested on polyester-cotton blended fabrics, this insect repellent demonstrated > 90% repellency. Its performance not only surpassed that of untreated fabrics but also outperformed traditional caprolactam-based repellents. The microencapsulated formulation also demonstrated sustained and effective mosquito-repelling performance. Javier et al. employed the Hansen solubility parameter model to design a physical gel composed of the insect repellent N,N-diethyl-m-toluamide (DEET) and the modified acrylic copolymer poly(acrylonitrile-co-vinyl chloride) (P(AN-VC)), serving as the polymer matrix. The resulting P(AN-VC)/DEET composite demonstrated sustained mosquito repellency for > 6 months due to both its high repellent loading capacity and the polymer matrix’s ability to inhibit adsorption. This system outperformed all previously reported repellent delivery materials in term of longevity and efficacy [182]. SiO_2_-IR3535 mosquito repellent nanocapsules, prepared by encapsulating the repellent IR3535 with silica-based nanostructures, have been shown to impart durable mosquito-repellent properties to treated textile fibers [183]. In a related study, a nanoemulsion finishing agent incorporating betel nut and clove essential oils, encapsulated using cationic guar gum as the carrier, achieved an encapsulation efficiency > 50% and enabled controlled release for up to 150 h. When applied to cotton and polyester fabrics, the treated textile achieves 98–100% repellency against *Aedes aegypti* mosquitoes after one wash-and-heat cycle and maintained 36%–40% repellency even after five such cycles [184]. Additionally, a sustained-release mosquito repellent system was developed using DEET as the active ingredient combined with porous silica or polyester adsorbents surface modified with hydroxyl-siloxane groups. This formulation significantly extends the repellent efficacy of DEET at conventional concentrations while simultaneously reducing skin irritation [185]. Additionally, nanoemulsions containing thymol, carvacrol, and cinnamaldehyde were prepared using a self-emulsification method. Following gelation with hydroxypropyl methylcellulose (HPMC), a 3% w/v thymol-based nanogel was obtained. Similarly, a 3% w/v carvacrol-based nanogel demonstrated mosquito-repellent protection against *Aedes aegypti* for 250 ± 34 min. This performance surpassed that of a commercial formulation containing 40% w/v DEET. These findings highlight the potential of nanogel systems incorporating natural compounds as promising alternatives for next-generation mosquito repellent [186].

**Fig. 24:**
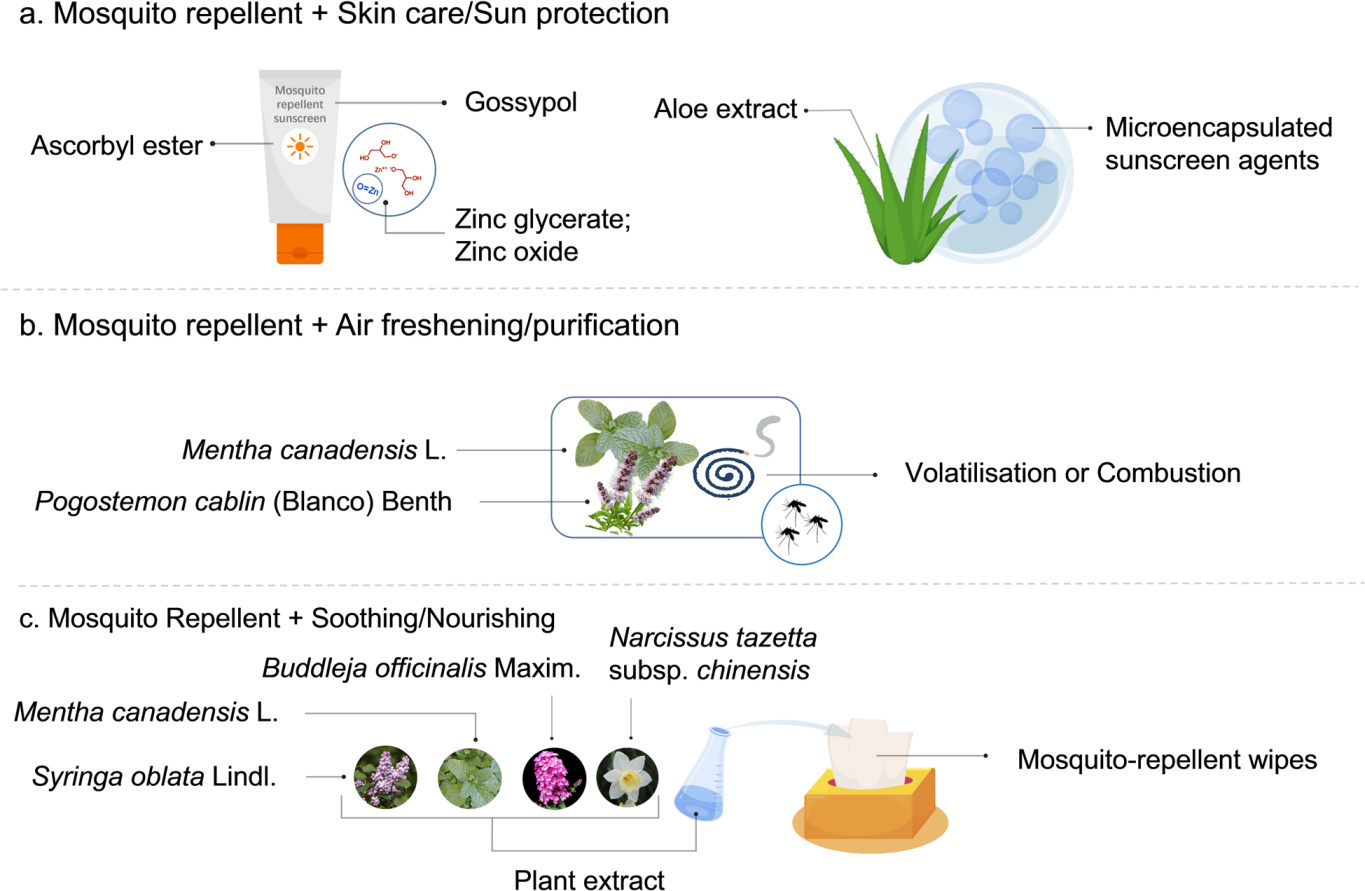
Multifunctional composite mosquito-repellent formulation design. **a** Mosquito repellent + skin care/sun protection. **b** Mosquito repellent + air freshening/purification. Images were taken from Pixabay (https://pixabay.com). **c** Mosquito repellent + soothing/nourishing. Images were created with BioGDP.com[20] and provided by Pixabay (https://pixabay.com)

Additionally, to address consumers’ extended needs for multi-scenario protection and skin care, synergistic functional ingredient technology enables formulations to deliver multiple benefits within a single formulation (Fig. 24a, b, c). A multifunctional protective cream combining sun protection, skin repair and mosquito repellent. By synergistically blending natural ingredients such as gossypol and ascorbyl esters with zinc-based additives within a single formulation, this product delivers broad-spectrum sun protection and damage repair while repelling mosquitoes and providing antiviral benefits [187]. In another application, a mosquito-repellent sunscreen designated for children was formulated by blending glyceryl stearate, aloe leaf juice, and encapsulated sunscreen agents. This multifunctional formulation provides effective, non-irritating sun protection while simultaneously delivering long-lasting mosquito repellency [188]. Some mosquito repellents improve or freshen the surrounding air environment while repelling insects. Zhao Fengyan et al. [189] employed an orthogonal experimental design in combination with an enzymatic-assisted steam distillation method to extract volatile oils from mugwort. These oils were then subsequently blended with honeysuckle extract, peppermint extract, and other natural components to develop a compound mugwort-based mosquito repellent. The product met national standards for sensory quality, physicochemical properties, and skin irritation and was classified as having Class B mosquito repellency efficacy as defined by the National Standard. Meanwhile, traditional Chinese herbal mosquito repellent techniques have been integrated into modern manufacturing processes to establish a distinctive approach. Herbal mosquito-repellent incense sticks, formulated with medicinal herbs, such as patchouli (*Pogostemon cablin* [Blanco] Benth.) and peppermint (*Mentha canadensis* L.), are manufactured using distilled water extraction and low-temperature drying techniques. Upon combustion, these sticks release the aromatic herbal volatile that not only repels mosquitoes but also purifys the surrounding air [190]. Modern mosquito repellents can provide immediate care or soothing effects for the skin. A mosquito-repellent wipe was developed using soft, breathable, and highly absorbent spunlace nonwoven fabric as the substrate. The fabric is impregnated with a compound solution containing 1–5% plant extracts of peppermint (*Mentha canadensis* L.), clove (*Syringa oblata* Lindl.), bee balm (*Buddleja officinalis* Maxim.), and *Narcissus*, with the remainder comprising purified water. Its natural ingredients provide effective mosquito repellency while being gentle on the skin. Additionally, the mint extract imparts a cooling sensation and provides relieve from pruritus [191].

## Regulatory framework and technological pathways of mosquito-repellent development in China

Regarding repellent policy, China is promoting the industrialization of mosquito vector control through a regulatory framework comprising national standards and regional policies. At the product management level, repellents are classified under pesticide regulations, with clear concentration limits for DEET products (2–15%) and specific control requirements for children’s formulations [192]. Strictly speaking, any new mosquito repellent or insecticide product that is marketed without appropriate certification or bears non-compliant labeling is classified as a counterfeit pesticide and is prohibited from sale [193]. Regarding efficacy evaluation, the national standard GB/T 13917.9-2009 “Indoor Efficacy Testing and Evaluation of Insecticides for Pesticide Registration-Part 9: Repellents” establishes a graded evaluation framework [194]. It specifies core performance indicators: Grade A products must provide effective protection for ≥ 6 h, while Grade B products must provide ≥ 4 h of protection. These criteria serve as mandatory benchmarks for evaluating the efficacy of repellent formulations. The aforementioned standards work in tandem with specialized standards such as T/CPCIF 0043-2020, Determination of 7 Insect Repellents Including DEET and DEET Equivalents in Mosquito Repellent Products [195], and T/ZZB 2850-2022 Plant Essential Oil Mosquito Repellent Sprays [196]. This coordinated regulatory system not only standardizes market practices but also guides product research and development toward greater efficiency, safety, and environmental sustainability. At the regional policy level, local authorities have introduced supplementary requirements tailored to their specific geographic, ecological, and public health needs, thereby reinforcing the effectiveness of mosquito vector control programs. For instance, Yunnan’s “Chemical-Free Mosquito Repellent Demonstration Zone” sets a DEET concentration limit of ≤ 10%, significantly stricter than the national standard for adult products [197]. In Guangdong Province, specialized testing standards have been introduced for mosquito-repellent products intended for use by mothers and infants—a particularly sensitive population group. These standards include additional screening protocols for endocrine-disruption compounds, with testing requirements surpassing those of national guidelines, thereby elevating the safety threshold for products targeting vulnerable populations [198]. In terms of international trade management, China has implemented strict import and export control procedures for pesticide-related products. All mosquito-repellent products exported to China or sold within China must bear Chinese-language labels in full compliance with the Measures for the Administration of Pesticide Labels and Instructions. Additionally, importers are required to apply to both the Ministry of Agriculture and Rural Affairs and the General Administration of Customs to obtain a Pesticide Import/Export Registration Management Release Notice [199, 200]. Notably, regulatory requirements for insect repellents vary significantly across different countries and regions, owing to differences in mosquito species, epidemiological profiles, environmental protection priorities, and public health considerations. For instance, the United States Environmental Protection Agency (EPA) [201] limits DEET concentrations to 10–30% (≤ 10% for children’s products) and mandates the submission of long-term ecotoxicological data for product registration. Nationwide and regional regulations in China have increasingly steered the technological trajectory and market structure of mosquito-repellent products. At the national level, restrictions on DEET concentrations, rigorous standards for children-specific formulations, and standardized labeling requirements have compelled manufacturers to reformulate their products and enhance ingredient transparency. Regionally, targeted policies—such as Yunnan’s cap on DEET content (≤ 10%) and Guangdong’s additional screening for endocrine-disrupting chemicals in mother-infant repellent products—have further accelerated the industry’s shift toward plant-derived, low-toxicity, and controlled-release formulations. Consequently, regulatory pressure has emerged as a key driver of innovation, propelling China’s domestic mosquito repellent industry toward the development of safer, more diversified, and higher-quality solutions.

Propelled by policy-driven pressures for the transformation and upgrading of China’s mosquito repellent industry, the domestic market has broken free from its long-standing reliance on original equipment manufacturing (OEM) and product imitation. It is progressively shifting toward technological innovation and the innovative utilization of indigenous resources—thus fostering the development of core competitive advantages. China’s geographically diverse landscape has nurtured a rich reserve of regionally distinctive mosquito-repellent plant resources, which serves as a pivotal foundation for driving industry-wide innovation. For instance, the dried tangerine peel trees (*Citrus reticulata* ‘Chachi’) native to Xinhui in southern Guangdong yield fruit peels rich in limonene and linalool—two bioactive compounds that demonstrate potent mosquito-repellent properties [202]. Northern mugwort (*Artemisia vulgaris* var. *mongolica*), whose leaves contain eucalyptol, allows for straightforward extraction of its insect-repellent components. In the southwestern ethnic minority regions of China, the Miao people have preserved a traditional practice of using “Artemisia branches and leaves for mosquito repellency”—either by crafting them into sachets or boiling them to produce a sprayable solution [203]. Such plants have transitioned from traditional folk resources to core indigenous ingredients for mosquito repellents. Current market offerings include mosquito-repellent patches and wristbands formulated with artemisia extract—products specifically tailored for sensitive populations such as pregnant women and infants. Furthermore, dried tangerine peel is frequently blended with mugwort and lemongrass to produce traditional Chinese-style mosquito-repellent incense sticks. When burned, these incense sticks release an aroma that not only repels mosquitoes but also carries a subtle sweet citrus note. While Chinese enterprises once depended heavily on imported raw materials and formulations, domestic technological innovation now facilitates the high-value conversion of indigenous plant resources. For instance, supercritical carbon dioxide (CO_2_) extraction technology enhances the purity of dried tangerine peel essential oil, with limonene—its primary active component—reaching a content of 51%. When applied to fabrics, this essential oil achieves a mosquito repellency rate of > 90% [204]. Meanwhile, microencapsulation technology effectively mitigates the volatility of mugwort oil—an essential technical solution to overcome the core challenge of maintaining the stability of plant-derived mosquito-repellent active ingredients [205]. This development not only fills the gap in consumer demand for natural mosquito repellents but also aligns with the rapid expansion of China’s natural mosquito repellent market over the past 5 years. Products derived from dried tangerine peel, *Artemisia annua*, and mugwort now account for a substantial share of this market. Concurrently, under policy guidance, research focused on indigenous plant resources has advanced with the establishment of standardized methodologies and data frameworks that provide robust technical support for repellent product development. This, in turn, further solidifies the foundational basis for innovation within the industry. The Institute of Zoology, Chinese Academy of Sciences (CAS) [206], has established an “electrophysiological screening-behavioral validation” integrated system. This methodology utilizes electroantennogram (EAG) analysis to isolate mosquito-repellent active compounds, verifies their efficacy via Y-tube olfactometer assays, and clarifies the interaction mechanisms between essential oils derived from traditional Chinese medicines (TCMs) and mosquito olfactory receptors. Furthermore, the institute has developed an “activity-environment-effect” data framework for grape seed molecule research, which meets the requirements for quantitative structure-activity relationship (QSAR) modeling of DEET alternatives. This system and framework collectively provide experimental evidence to support the prediction of both the repellent activity and environmental adaptability of novel compounds, thereby accelerating the development of high-performance synthetic mosquito repellents.

Therefore, driven by regulatory pressures, China’s mosquito repellent industry has successfully moved beyond its imitative OEM model. Through innovations in in-depth integration of indigenous technological and in regionally distinctive local resources, the industry has not only expanded its market presence and improved product quality but also established core competitiveness—ultimately propelling the entire sector into a new phase of high-quality development.

## Conclusions

The prevention and control of mosquito-borne diseases constitute a vital component of global public health systems, dedicated to safeguarding human well-being. The synergistic application of mechanical, biological, and chemical control methods has become a key support for constructing comprehensive mosquito control systems. Among these, mechanical control, serving as the fundamental means for physically blocking mosquito transmission, plays an irreplaceable role in interrupting the transmission chain of mosquito-borne diseases. However, its application needs to be further refined to enhance operational precision and ecological compatibility, thereby laying a robust foundation for the efficient deployment of biological and chemical control. Biological control, characterized by its environmentally sustainable attributes, has achieved a series of breakthroughs in areas such as microbial agent development and gene-editing technology applications. Nevertheless, it still faces critical bottlenecks that need to be addressed, including limited storage stability, the absence of comprehensive safety risk regulatory systems, and inadequate adaption to localized ecological conditions.

In the realm of chemical control, mosquito repellents constitute a vital measure to prevent the transmission of mosquito-borne diseases and hold significant implications for public health protection. Given their crucial role, it is imperative to further advance research, development, and application of mosquito repellents through interdisciplinary innovation and evidence-based formulation strategies. Strengthening these efforts will contribute not only to enhancing individual protection but also to supporting broader vector control initiatives and improving global health resilience. With the continuous increase in people’s consumption level, and aiming to improve the quality of life and of the environment, the demand for repellents will continue to increase, especially those suitable for children and pregnant women, and more attention will be paid to the environmental protection, safety, and versatility of mosquito-repellent products. Many mosquito-repellent products are not suitable for direct use in children or pregnant women because of potential safety concerns. However, these two groups are particularly vulnerable to mosquito-borne viruses, highlighting the urgent need to develop repellents specifically designed for their use. In the long term, the consumer market for mosquito repellents is expected to continue expanding. Therefore, the development of novel repellent products that are safe, environmentally friendly, gentle, and convenient to use is essential for driving sustained growth in the mosquito repellent industry. Researchers can build upon existing compounds with proven repellent efficacy by employing computer-aided design to develop novel synthetic molecules and construct new libraries of mosquito repellent candidates, thereby enriching the diversity of repellent agents and enabling optimal compound selection based on specific application needs. For natural repellents, further exploration is warranted to identify safe, effective, and environmentally friendly plant-derived compounds. To overcome limitations such as high volatility and short duration of action, advanced technologies and novel formulations should be employed to optimize performance. This includes the development of various practical dosage forms, reduction of dermal absorption, and extension of repellent efficacy. Furthermore, the toxicological profiles and mechanisms of action of most repellents on humans remain insufficiently understood. Therefore, it is essential to strengthen research on the interactions among mosquito physiology, behavior, and repellent agents to elucidate their modes of action. Such understanding is critical to guiding the rational design and development of next-generation mosquito repellents.

Currently, China’s mosquito-borne disease prevention and control efforts are progressively aligning with global trends in integrated mosquito management. In the future, it will be guided by the core principles of “green, precise, and intelligent” control, with a focus on promoting the technological iteration and upgrading of locally sourced plant mosquito repellents and smart monitoring devices. In terms of system construction, although domestic policies and regulations are continuously improving, notable compliance gaps persist within the mosquito-repellent product market. These gaps pose significant barriers to the high-quality development of the industry and constrain the overall effectiveness of mosquito-borne disease prevention and control. Existing studies have identified a number of prominent issues in the current market [207]. First, labeling irregularities are widespread, including incomplete product information, and discrepancies between declared ingredients and actual content exist, hindering consumers’ ability to make informed, science-based decisions. Second, safety risks persist within product formulations; some emerging products illegally contain unverified ingredients, posing potential risks that are difficult to regulate. Third, misleading marketing practices are prevalent, with some enterprises exaggerating product efficacy and concealing usage limitations, thereby encouaging consumers to make purchasing and usage decisions that do not align with scientific guidance. Considering these challenges, it is essential to further strengthen regulatory oversight of mosquito-repellent products. Efforts should focus on establishing a coordinated and unified mosquito-borne disease control and corresponding product supervision frameworks. By standardizing market practices and promoting industrial upgrading, these measures will contribute to establishing a domestic public health security protection system, providing a practical reference for global efforts to prevent and control mosquito-borne diseases.

In summary, effective mosquito-borne disease prevention and control requires integrated application and coordinated advancement of mechanical, biological, and chemical approaches. These measures must work in tandem to block transmission routes at the source, thereby substantially reducing the risk of disease spread. To ensure practical efficacy, interventions must be tailored to local ecological conditions, epidemiological features, and governance frameworks. At the same time, it is imperative to align with the global trend in integrated vector management by benchmarking against advanced technologies and international regulatory standards, thereby continuously enhancing the overall effectiveness of mosquito-borne disease control systems. This review aimed to contribute to this goal by systematically summarizing integrated control strategies, with a particular focus on repellent development, application, and Chinese practices. However, it is important to acknowledge its limitations. First, the research perspective is heavily weighted toward the Chinese context, with relatively limited exploration of localized experiences and challenges in other significant endemic regions. Consequently, the global applicability of the conclusions requires further validation. Second, analyses of long-term ecological risks, bottlenecks in large-scale implementation, and socioeconomic feasibility for certain cutting-edge technologies—such as genetically modified mosquitoes and novel delivery systems—remain inadequate. Furthermore, potential risks such as a repellent's environmental persistence and mosquito resistance evolution, alongside integration pathways for interdisciplinary approaches—including behavioral interventions and digital technologies—within integrated management systems warrant deeper exploration in future research. These limitations point to the need for continued efforts in broader global comparative studies, more comprehensive techno-economic-ecological assessments, and enhanced interdisciplinary collaboration.

## Data Availability

No datasets were generated or analyzed during the current study.

## Abbreviations

KBR 3023: Sec-butyl 2-(2-hydroxyethyl)piperidine-1-carboxylate
IR3535: Ethyl butylacetylaminopropionate
SS220: (1S,2S)-2-methylpiperidine-3-cyclohexene-1-carboxamide
DMP: Dimethyl 1,2-phthalate
PMD: Para-menthane-3,8-diol
AI3-372201: -(2-cyclohexen-1-yl)carbonyl-2-methylpiperidine
EPA: United States Environmental Protection Agency
DEPA: N, N-diethylphenylacetamide
DPA: Diethylphenylacetamide
EOs: Essential oils
BioUD: Biological 2-Undecanone
USDA: United States Department of Agriculture
DRDE: Development establishment
US: United States
*Cx. quinquefasciatus*: *Culex quinquefasciatus*
*Ae. aegypti*: *Aedes aegypti*
*An. gambiae*: *Anopheles gambiae*
*Ae. albopictus*: *Aedes albopictus*
ORNs: Olfactory receptor neurons
CRS: Controlled release system
ORs: Olfactory receptors
OR: Odor receptor
Orco: Odor receptor co-receptor
OSNs: Olfactory sensory neurons
AgOR2: *Anopheles gambiae* olfactory receptor 2
AgOR8: *Anopheles gambiae* olfactory receptor 8
AgOrco: *Anopheles gambiae* odor receptor co-receptor
Ir40a: Ion receptor 40a
GMM: Genetically modified mosquito
SIT: Sterile insect technique
Bti: *Bacillus thuringiensis subsp. israelensis*
Bs: *Bacillus sphaericus*
OR: Insecticide-susceptible Orlando
PR: Pyrethroid-resistant Puerto Rico
MUP: Manufacture use product
EC: Emulsifiable concentrate
P(AN-VC): Poly(acrylonitrile-co-vinyl chloride)
HPMC: Hydroxypropyl methylcellulose
AgamOBP1: *Anopheles gambiae* odorant binding protein 1
CquiOR136: *Culex quinquefasciatus* odorant receptor 136
CquiOR32: *Culex quinquefasciatus* odorant receptor 32
QSAR: Quantitative structure-activity relationship
OEM: Original equipment manufacturing
CAS: Chinese Academy of Sciences
EAG: Electroantennogram
TCMs: Traditional Chinese medicines
